# Multilocus phylogenies reveal three new truffle-like taxa and the traces of interspecific hybridization in *Octaviania* (*Boletaceae*, *Boletales*)

**DOI:** 10.1186/s43008-021-00066-y

**Published:** 2021-06-11

**Authors:** Takamichi Orihara, Rosanne Healy, Adriana Corrales, Matthew E. Smith

**Affiliations:** 1 Kanagawa Prefectural Museum of Natural History, 499 Iryuda, Odawara, Kanagawa 250-0031 Japan; 2 Department of Plant Pathology, University of Florida, Gainesville, Florida 32611-0680 USA; 3 Centro de Investigaciones en Microbiología y Biotecnología-UR (CIMBIUR), Facultad de Ciencias Naturales, Universidad del Rosario, Bogotá, 111221 Colombia

**Keywords:** *Boletaceae*, Hypogeous fungi, Phylogeography, Sequestrate fungi, Systematics, 3 new taxa

## Abstract

Among many convergently evolved sequestrate fungal genera in *Boletaceae* (*Boletales*, *Basidiomycota*), the genus *Octaviania* is the most diverse. We recently collected many specimens of *Octaviania* subg. *Octaviania,* including several undescribed taxa, from Japan and the Americas. Here we describe two new species in subgenus *Octaviania*, *O. tenuipes* and *O. tomentosa*, from temperate to subtropical evergreen *Fagaceae* forests in Japan based on morphological observation and robust multilocus phylogenetic analyses (nrDNA ITS and partial large subunit [LSU], translation elongation factor 1-α gene [*TEF1*] and the largest subunit of RNA polymerase II gene [*RPB1*]). Based on specimens from the Americas as well as studies of the holotype, we also taxonomically re-evaluate *O. asterosperma* var. *potteri*. Our analysis suggests that *O. asterosperma* var. *potteri* is a distinct taxon within the subgenus *Octaviania* so we recognize this as *O. potteri* stat. nov. We unexpectedly collected *O. potteri* specimens from geographically widespread sites in the USA, Japan and Colombia. This is the first verified report of *Octaviania* from the South American continent. Our molecular analyses also revealed that the *RPB1* sequence of one *O. tenuipes* specimen was identical to that of a closely related species, *O. japonimontana*, and that one *O. potteri* specimen from Minnesota had an *RPB1* sequence of an unknown species of *O.* subg. *Octaviania*. Additionally, one *O. japonimontana* specimen had an unusually divergent *TEF1* sequence. Gene-tree comparison and phylogenetic network analysis of the multilocus dataset suggest that these heterogenous sequences are most likely the result of previous inter- and intra-specific hybridization. We hypothesize that frequent hybridization events in *Octaviania* may have promoted the high genetic and species diversity found within the genus.

**Supplementary Information:**

The online version contains supplementary material available at 10.1186/s43008-021-00066-y.

## INTRODUCTION

The *Boletaceae* (*Boletales, Basidiomycota*) is a large family that mostly consists of epigeous, mushroom-forming fungi. However, recent systematic studies have revealed a considerable number of sequestrate (i.e. truffle-like and secotioid) fungal lineages in the family that have evolved independently from boletoid mushrooms (e.g., Castellano et al. 2016; Desjardin et al. 2008, 2009; Lebel et al. 2012a, 2012b; Nuhn et al. 2013; Orihara et al. 2010, 2016a, 2016b; Orihara and Smith 2017; Smith et al. 2015, 2018; Sulzbacher et al. 2020; Vadthanarat et al. 2018; Wu et al. 2016). Although many sequestrate genera in *Boletaceae* comprise one or a few species, the genus *Octaviania* (orthographic variant: *Octavianina*), which belongs to the subfamily *Leccinoideae* (Wu et al. 2014), is exceptionally diverse and includes more than 25 truffle-like species (Orihara et al. 2012a; Paz et al. 2014, 2016).

The genus *Octaviania* is comprised of sequestrate, truffle-like species that have a marbled gleba and dextrinoid or non-amyloid basidiospores with coarse, conical to pyramidal ornamentation (Orihara et al. 2012a). Historically, the generic concept of *Octaviania* was unsettled and the genus was previously considered by some authors as a synonym of *Arcangeliella* (*Russulaceae*), *Hydnangium* (*Hydnangiaceae*). or *Melanogaster* (*Paxillaceae*). Pegler and Young (1979) provided evidence that *Octaviania* is distinct from those morphologically similar sequestrate genera and Orihara et al. (2012a) redefined the current generic concept of the genus. Orihara et al. (2012a) further divided the genus into three subgenera, *Octaviania*, *Fulvoglobus,* and *Parcaea*, based on multigene phylogenies and the morphology of basidiomata. Paz et al. (2016) reviewed the European species of *Octaviania* and critically examined the type species, *O. asterosperma*. They found that *O. asterosperma s. str.* has a pseudoparenchymatous peridium, which is one of the major characteristics of subg. *Fulvoglobus*. Accordingly, they concluded that the subg. *Fulvoglobus* introduced by Orihara et al. (2012a) should be synonymized with subg. *Octaviania* sensu Paz et al. (2016), and that the preceding subg. *Octaviania* sensu Orihara et al. (2012a) should be synonymized with subg. *Mutabiles*.

*Octaviania* subg. *Octaviania* sensu Paz et al. (2016), hereafter referred to as *Octaviania* subg. *Octaviania,* is characterized by cavities in the gleba filled with slightly viscid to dry, brown to blackish brown spore masses, and a peridium composed of inflated hyphae and isodiametric, pseudoparenchymatous cells. So far, the subgenus accommodates eight described species that are known only from the northern hemisphere (Orihara et al. 2012a; Paz et al. 2016). Orihara et al. (2012a) further suggested that there were at least two additional, taxonomically unsettled species (*Octaviania* sp. “E” from Japan and *Octaviania* sp. from North America). Since the publication of Orihara et al. (2012a), we have collected a number of additional specimens of *O.* subg. *Octaviania* from Japan, including the two species mentioned above. In addition, we collected basidiomes of *O.* subg. *Octaviania* from a *Quercus humboldtii* forest in Colombia, which constitutes the first known record of *Octaviania s. str*. from South America. Our primary objective is to clarify the phylogenetic and systematic positions of those taxonomically unsettled specimens in a robust phylogenetic framework. Here we propose two new species and one new taxonomic rank based on morphological observations and multilocus phylogenies. Furthermore, we found strong topological conflicts in some species of *O.* subg. *Octaviania* among gene trees. We therefore examined the cause of these conflicts using gene-tree comparisons and phylogenetic network analyses and discuss the possibility of inter- and intra-specific hybridization within the subgenus based on their ecology and phylogeography.

## METHODS

### Taxon sampling and morphological observation

Fresh basidiomes were collected throughout Japan, from eastern North America, and from Colombia. All collecting sites were dominated by *Fagaceae* trees (i.e., *Quercus, Castanopsis* or *Lithocarpus* spp.). After morphological observation, the basidiomes were air-dried or freeze-dried and then stored in sterile plastic bags. These specimens are deposited in Kanagawa Prefectural Museum of Natural History, Japan (KPM), Ada Hayden Herbarium, Iowa State University (ISC), Bell Museum of Natural History Herbarium Fungal Collection, University of Minnesota (MIN), Farlow Herbarium, Harvard University (FH), Florida Museum of Natural History Fungal Herbarium, University of Florida (FLAS), and the Oregon State University Herbarium (OSC). Other specimens were also obtained from KPM, FLAS, ISC, OSC, and the University of Michigan Herbarium (MICH).

For microscopy, hand-cut sections of fresh or dried specimens were mounted in water, 3% KOH or lacto-glycerol. To determine amyloidity of basidiospores, dried material was stained with Melzer’s reagent. Basidiospore dimensions (range of spore length, from the hilar appendage to the spore tip × spore width), their standard deviations (SD) and the length to width ratio (*Q*) were determined based on 50 random measurements unless otherwise mentioned. The 95% prediction intervals of basidiospore diameter are shown without parentheses in taxonomic descriptions. Both endpoints of the spore dimensions are shown in parentheses, but when the value is the same as the 95% prediction interval, only the latter is shown. Measurements include the hilar appendage but not the spore ornamentation or pedicel. Basidium sizes are presented as the range of the lengths × the range of the widths. Scanning electron microscopy (SEM) was performed with the HITACHI TM-4000Plus Tabletop Microscope (Hitachi High-Technologies, Japan). Small fragments of a dried gleba were excised and immersed in 8% ionic liquid (1-ethyl-3-methyl-imidazolium tetrafluoroborate) for conductive treatment (Yanaga et al. 2012) and were observed at 10–15 kV.

### DNA extraction, PCR amplification and sequencing

DNA was extracted from fresh or dried basidiomes using Indicating FTA Cards (Whatman International, Maidstone, UK) based on the protocol by Orihara et al. (2012a, 2012b). We also extracted genomic DNA from some basidiomes using the protocol of Izumitsu et al. (2012). PCR amplification of the ITS and the large subunit (LSU; 28S) of the nuclear ribosomal DNA (nrDNA), and *TEF1* followed Orihara et al. (2012a). For *RPB1* amplification, we used a newly designed primer set based on sequences of *Boletaceae* deposited in the International Nucleotide Sequence Databases (INSD). The new primers include forward primer RPB1-TO-Bf (5′- AAGGCYGATATYGTGAGTC - 3′), which is located in the intron A between domains A and B of *RPB1*, reverse primer RPB1-TO-Br (5′- GCTTTGATGATRTCYCC - 3′), and reverse primer RPB1-TO-Br2 (5′- ARGCYTTGATRATRTCYCC- 3′). Both of the reverse primers are located in the conserved (exon) domain C. These primer pairs target an 850–1100 bp amplicon which spans the region between primer RPB1-Bf (Nuhn et al. 2013) and primer RPB1-Cr (Matheny et al. 2002). The PCR amplification of *RPB1* was performed using the following procedure: initial incubation at 95 °C for 10 min; subsequent step of 30 cycles at 94 °C for 30 s, 53 °C for 60 s, and 72 °C for 90 s, followed by 13 cycles at 94 °C for 30 s, 52 °C for 60 s, and 72 °C for 90 s; a final elongation step at 72 °C for 7 min. Unidirectional sequencing of the PCR products in the forward and reverse directions were completed according to Orihara et al. (2012a). Sequences were edited and assembled with Sequence Scanner v. 1.0 (Applied Biosystems, Foster City, CA, USA), BioEdit version 7.0.9 (Hall 1999) and SeaView version 4 (Galtier et al. 1996). A total of 178 newly obtained sequences were deposited in INSD (Table 1).

**Table 1:** Specimens and sequences used for the molecular phylogenetic analyses Sequences newly generated for this study are designated in bold. Specimens with an asterisk (*) correspond to the sequences with an asterisk in the same taxa. Specimens used only for supplementary nLSU phylogenetic analyses (Fig. S1) are designated with double asterisks (**)

Taxon	Voucher No.	Locality	ITS	28S	*TEF*1	*RPB*1
*Chamonixia caespitosa*	KPM-NC 18071	Japan, Nagano Pref., Mts. Yatsugatake	KP222908	**MT812734**	**MT874820**	**MT868871**
*Chamonixia caespitosa*	KRA F-2013-38	Poland, Gorce Mts.	KT001255	**MT812735**	**MT874821**	**MT868872**
*Leccinellum* aff. *griseum*	KPM-NC 17831	Japan, Hyogo Pref., Uwano Town	KC552008	JN378508	JN378449	**MT868816**
*Leccinellum* aff. *griseum*	KPM-NC 24518 (MO455)	Japan, Tochigi Pref., Nikko City	–	**MT812705**	**MT874790**	**MT868817**
*Leccinellum albellum*	KUO-07241101/FLAS-F-61741*	USA, North Carolina; *USA, North Carolina, Buncombe Co.	MH488723*	MK601746	MK721100	**–**
*Leccinellum crocipodium s.l.*	KPM-NC 18041	Japan, Tottori Pref., Yazu Town	–	KC552053	KC552094	**MT868818**
*Leccinellum corsicum*	Buf4507	USA	–	KF030347	KF030435	KF030389
*Leccinellum quercophilum*	M Kuo 07120801	USA, Illinois, Coles Co., Charleston	KC691207	KC691208	MK721178	–
*Leccinellum* sp.	HKAS 50221	China	JQ928612	JQ928624	JQ928583	JQ928593
*Leccinum* aff. *aurantiacum*	HKAS 57390	China, Yunnan Prov.	–	JQ928625	JQ928581	JQ928591
*Leccinum* aff. *schistophilum*	KPM-NC 17841	Japan, Hyogo Pref., Uwano Town	KC552011	KC552055	KC552096	**MT868874**
*Leccinum monticola*	HKAS 76669	China, Jilin Province, Yanbian	–	KF112443	KF112249	KF112592
*Leccinum quercinum*	HKAS 63502	China, Yunnan Province, Lijiang	–	KF112444	KF112250	KF112593
*Leccinum scabrum*	KPM-NC 17840	UK, Scotland, Burn O′ Vat	KC552012	JN378515	JN378455	**MT868875**
*Leccinum subradicatum*	KPM-NC 24518	Japan, Tochigi Pref., Nikko City	**MT934814**	**MT812736**	**MT874822**	**MT868873**
*Leccinum variicolor*	HKAS 57758	China, Yunnan Province, Lijiang	–	KF112445	KF112251	KF112591
*Leccinum versipelle*	KPM-NC 18036	UK, Scotland	–	**MT812737**	**MT874823**	**MT868876**
*Leccinum violaceotinctum*	CFMR BZ-1676 BOS-327	Belize	MN250203	MK601779	MK721133	–
*Leccinum violaceotinctum*	CFMR BZ-3169 BOS-616	Belize	MN250215	MK601780	MK721134	–
*Octaviania tenuipes* sp. nov.	KPM-NC 28187	Japan, Tokyo Met., Hachioji City, Mt. Takao	–	**MT812719**	**MT874805**	**MT868852**
*Octaviania tenuipes* sp. nov.	KPM-NC 27956	Japan, Chiba Pref., Katsuura City, Okitsu	**MT934803**	**MT812720**	**MT874806**	**MT868853**
*Octaviania tenuipes* sp. nov.	KPM-NC 27957	Kanagawa Pref., Hakone Town, Hakone-Yumoto	–	**MT812721**	**MT874807**	**–**
*Octaviania tenuipes* sp. nov.	KPM-NC 25370	Kanagawa Pref., Odawara City, Iryuda	**MT934804**	**MT812722**	**MT874808**	**MT868854**
*Octaviania tenuipes* sp. nov.	KPM-NC 26008	Japan, Tokyo Met., Hachijo Isl.	**MT934805**	**MT812723**	**MT874809**	**MT868855**
*Octaviania tenuipes* sp. nov.	KPM-NC 27960	Japan, Miyazaki Pref., Miyazaki City	–	**MT812724**	**MT874810**	**MT868856**
*Octaviania tenuipes* sp. nov.	KPM-NC 27965	Japan, Miyazaki Pref., Nichinan City	**MT934806**	**MT812725**	**MT874811**	**MT868857**
*Octaviania tenuipes* sp. nov.	KPM-NC 27968	Japan, Kagoshima Pref., Tarumizu City, Mt. Tohken	**MT934807**	**MT812726**	**MT874812**	**MT868858**
*Octaviania tenuipes* sp. nov.	KPM-NC 27972	Japan, Miyazaki Pref., Aya Town	**MT934808**	**MT812727**	**MT874813**	**MT868859**
*Octaviania tenuipes* sp. nov.	KPM-NC 24889	Japan, Kagoshima Pref., Tanegashima Isl.	**MT934809**	**MT812728**	**MT874814**	**MT868860**
*Octaviania tenuipes* sp. nov.	KPM-NC 24891	Japan, Kagoshima Pref., Tanegashima Isl.	**MT934810**	**MT812729**	**MT874815**	**MT868861**
*Octaviania tenuipes* sp. nov.	KPM-NC 27932	Japan, Kagoshima Pref., Yakushima Isl.	–	**MT812730**	**MT874816**	**MT868862**
*Octaviania tenuipes* sp. nov.	KPM-NC 17813	Japan, Kagoshima Pref., Amami-oshima Isl.	JQ619176	JN378487	JN378429	**MT868863**
*Octaviania tomentosa* sp. nov.	KPM-NC 27955	Japan, Kanagawa Pref., Minamiashigara City	**MT934797**	**MT812713**	**MT874799**	**MT868842**
*Octaviania tomentosa* sp. nov.	KPM-NC 27945	Japan, Kanagawa Pref., Minamiashigara City	**MT934798**	**MT812714**	**MT874800**	**MT868843**
*Octaviania tomentosa* sp. nov.	KPM-NC 27952	Japan, Tochigi Pref., Sano City, Mt. Karasawa	**MT934799**	**MT812715**	**MT874801**	**MT868844**
*Octaviania tomentosa* sp. nov.	KPM-NC 27954	Japan, Ibaraki Pref., Kasama City, Mt. Sashiro	**MT934800**	**MT812716**	**MT874802**	–
*Octaviania tomentosa* sp. nov.	KPM-NC 23934	Japan, Kagoshima Pref., Amami-oshima Isl., Uken-son Village	**MT934796**	**MT812712**	**MT874798**	**MT868841**
*Octaviania asterosperma* var. *potteri* *= O. potteri* stat. nov.	OSC 131925	USA, Florida, Wakulla Co., Skipper Bay road, St Marks NW refuge.	**MT934792**	JN378499	JN378441	**MT868835**
*Octaviania asterosperma* var. *potteri* *= O. potteri* stat. nov.	KPM-NC 17827 (RH30)	USA, Iowa, Story County, Ames, YMCA woods	–	JN378500	JN378442	**MT868836**
*Octaviania asterosperma* var. *potteri* *= O. potteri* stat. nov.	KPM-NC 18032	Japan, Hokkaido, Tomakomai City	**MT934795**	**MT812710**	**MT874796**	**MT868840**
*Octaviania asterosperma* var. *potteri* *= O. potteri* stat. nov.	KPM-NC 17828 (RH1181)	USA, Minnesota, Fillmore County, Forestville State Park.	**MT934793**	JN378501	JN378443	**No. 1 (seq1): MT868837** **No. 2 (seq2): MT868838**
*Octaviania asterosperma* var. *potteri* *= O. potteri* stat. nov.	HUA 222100 (AC-1036)	Colombia, Cundinamarca Province	**MT934794**	**MT812711**	**MT874797**	**MT868839**
*Octaviania potteri* (registered as “*Octaviania asterosperma*”)**	FH-284316 (RH3)	USA, Iowa	–	**MK601795**	–	–
*Octaviania durianelloides*	KPM-NC 17829	Japan, Kanagawa, Minamiashigara City	JQ619177	JQ619188	KJ001079	**MT868865**
*Octaviania durianelloides*	KPM-NC 18031	Japan, Hokkaido, Tomakomai City	**MT934811**	**MT812731**	**MT874817**	**MT868864**
*Octaviania durianelloides*	KPM-NC 28183	Japan, Yamaguchi Pref., Mt. Sobagatake	**MT934812**	**MT812732**	**MT874818**	**MT868866**
*Octaviania durianelloides*	KPM-NC 27371	Japan, Kanagawa, Odawara City, Kuno	**MT934813**	**MT812733**	**MT874819**	**MT868867**
*Octaviania hesperi*	KPM-NC 17792	Japan, Tokyo, Hachioji City	–	JN378479	JN378421	**MT868832**
*Octaviania hesperi*	KPM-NC 17793	Japan, Kanagawa Pref., Zushi City	JQ619173	JN378480	JN378422	**MT868833**
*Octaviania hesperi*	KPM-NC 28189	Japan, Kanagawa Pref., Hayama-mati	**MT934791**	**MT812709**	**MT874795**	**MT868834**
*Octaviania japonimontana*	KPM-NC 17798	Japan, Tottori Pref., Kofu Town, Kagamiganaru	–	JN378482	JN378424	**MT868845**
*Octaviania japonimontana*	KPM-NC 17797	Japan, Akita Pref., near Lake Towada	JQ619174	JN378483	JN378425	**MT868846**
*Octaviania japonimontana*	KPM-NC 17806	Japan, Tottori Pref., Mt. Daisen	–	JN378484	JN378426	**MT868847**
*Octaviania japonimontana*	KPM-NC 17810	Japan, Tottori Pref., Yazu Town	JQ619175	JN378485	JN378427	**MT868848**
*Octaviania japonimontana*	KPM-NC 17812	Japan, Okayama Pref., Kagamino Town	–	JN378486	JN378428	**MT868849**
*Octaviania japonimontana*	KPM-NC 27622	Japan, Kanagawa Pref., Tanzawa Mountains	**MT934801**	**MT812717**	**MT874803**	**MT868850**
*Octaviania japonimontana*	KPM-NC 27623	Japan, Kanagawa Pref., Tanzawa Mountains	**MT934802**	**MT812718**	**MT874804**	**MT868851**
*Octaviania kobayasii*	KPM-NC 17785	Japan, Nara Pref., Mt. Kasuga	JQ619170	JN378478	JN378420	**MT868829**
*Octaviania kobayasii*	KPM-NC 17783	Japan, Kyoto Pref., Uji City	JQ619171	JN378477	JN378419	**MT868830**
*Octaviania kobayasii*	KPM-NC 28188	Japan, Kanagawa Pref., Yokohama City, Minato-ku	**MT934790**	**MT812708**	**MT874794**	**MT868831**
*Octaviania etchuensis*	KPM-NC 17822	Japan, Toyama Pref., Nakashingawa-gun, Teteyama Town	JQ619182	JN378492	JN378433	**MT868870**
*Octaviania yaeyamaensis*	KPM-NC 17818	Japan, Okinawa Pref., Ishigaki Isl.	JQ619179	JN378490	JN378431	**MT868868**
*Octaviania yaeyamaensis*	KPM-NC 17819	Japan, Okinawa Pref., Ishigaki Isl.	JQ619180	JN378491	JN378432	**MT868869**
Octaviania asterosperma s. str.**	IC1091316	Spain, Cantabria	–	KX756591	–	**–**
Octaviania arbucalensis**	AH-43987	Spain, Zamora	–	KF154254	–	**–**
*Octaviania nonae*	KPM-NC 17748	Japan, Kagoshima Pref., Amami-oshima	JN257985	JN378459	JN378403	**MT868819**
*Octaviania nonae*	KPM-NC 17752	Japan, Hiroshima Pref., Hiroshima City, Higashi-ku	JN257989	JN378463	JN378407	**MT868820**
*Octaviania decimae*	KPM-NC 17763	Japan, Kyoto Pref., Mt. Hiei,	JN257991	JN378465	JN378409	**MT868821**
*Octaviania celatifilia*	KPM-NC 24872	Japan, Kagoshima Pref., Takakuma Ravine	**MT934785**	**MT812706**	**MT874791**	**MT868823**
*Octaviania mortae*	KPM-NC 17771	Japan, Kyoto Pref., Nanzen-ji Shrine	JN257995	JN378471	JN378414	**MT868822**
*Octaviania asahimontana*	KPM-NC 17824	Japan, Hokkaido, Mts. Daisetsu	JQ619178	JN378489	JN378430	**MT868828**
*Octaviania cyanescens*	OSC 58498	Canada, British Columbia, Vancouver Island	**MT934789**	JN378503	JN378439	**MT868827**
*Octaviania depauperata* var. depauperata**	JMV951116–2	Spain, Cataluña	**–**	KX756589	–	**–**
*Octaviania depauperata* var. laurarum**	IC24081315	Spain, Cantabria	**–**	KX756587	–	**–**
*Octaviania lutea*	AQUI 3899	Italy, Provincia L’Aquila, Comune di Cappadocia	–	KC552052	KC552093	**MT868825**
*Octaviania mutabilis*	KRA F-2012-99	Poland, Beskid Niski Mts.	**MT934787**	**MT812707**	**MT874793**	**MT868826**
Octaviania mutabilis**	IC14081321	Spain, Cantabria	**–**	KX756594	–	**–**
*Octaviania tasmanica*	MEL 2341996	Australia, Tasmania	**MT934786**	JN378495	**MT874792** ← JN378436	**MT868824**
*Octaviania zelleri*	MES270	USA, Maine, Tunk Lake, off route 182	**MT934788**	JN378498	JN378440	**–**
*Rossbeevera bispora*	KPM-NC 28186	China, Guangdong Province, Dinghu District	**MT934784**	**MT812704**	**MT874788**	**MT868814**
*Rossbeevera cryptocyanea*	KPM-NC 26877	Japan, Okinawa Pref., Kume-jima Isl.	**MT934783**	**MT812703**	**MT874787**	**MT868813**
*Rossbeevera eucyanea*	KPM-NC 28182	Japan, Yamaguchi Pref., Mt. Sobagatake	**MT934782**	**MT812702**	**MT874786**	**MT868812**
*Rossbeevera griseovelutina*	TNS-F-36990	Japan, Hyogo Pref.	HQ693876	HQ693881	KC552074	**MT868810**
*Rossbeevera griseovelutina*	TNS-F-36991	Japan, Okayama Pref.	KC551985	KC552032	KC552077	**MT868811**
*Rossbeevera pachydermis*	KPM-NC 23336	New Zealand, NZ North Isl., Te Urewera National Park	KJ001088	KJ001095	KP222912	**MT868809**
*Rossbeevera paracyanea*	KPM-NC 18087	Japan, Nara Pref., near Mt. Kasuga	KJ001086	KJ001100	**MT874789** ← KJ001082	**MT868815**
*Rossbeevera vittatispora*	MEL 2128491	Australia, New South Wales	**MT934781**	KX685725	KX685719	**MT868808**
*Rossbeevera vittatispora*	MEL 2329434	Australia, Victoria, Midlands	KJ001084	KJ001097	KJ001075	**MT868807**
*Rossbeevera yunnanensis*	KPM-NC 17850	China, Yunnan Prov., Chu Xang Pref., Mt. Zi Xi	KC551990	JN979437	KC552080	**MT868806**
*Turmalinea mesomorpha* subsp. *mesomorpha*	KPM-NC 18014	Japan, Iwate Pref., Appi-Kogen	KC552000	KC552048	KC552091	**MT868804**
*Turmalinea mesomorpha* subsp. *sordida*	KPM-NC 17743	Japan, Ehime Pref., Matsuyama City, Mt. Takanawa	KC552002	KC552050	KJ001078	**MT868805**
*Turmalinea persicina*	KPM-NC 18001	Japan, Kyoto Pref., Iwakura	KC551991	KC552038	KC552082	**MT868802**
*Turmalinea yuwanensis*	KPM-NC 18011	Japan, Kagoshima Pref., Amami-Ohshima Isl.	KC551998	KC552046	KC552089	**MT868803**
*Retiboletus fuscus*	HKAS 59460	China, Yunnan Prov.	JQ928613	JQ928626	JQ928580	JQ928590
*Retiboletus griseus*	Both sn	USA, New York	–	KF030308	KF030414	KF030373
*Borofutus dhakanus*	HKAS 73792	Bangladesh, Dhaka Division, Gazipur, Bhawal National Park	JQ928607	JQ928617	JQ928575	JQ928587
*Spongiforma thailandica*	DED 7873	Thailand, Nakorn Nayok Province, Khao Yai National Park	EU685113	EU685108	KF030436	KF030387
*Tylocinum griseolum*	HKAS 50281/HKAS 50209*	China, Yunnan Prov., Dadugang Town	–	KF112451	KF112284	KT990919*
*Spongispora temasekensis*	HKAS 101385	Singapore, Singapore Botanic Gardens	MG979395	MG672512	MG674377	MG979393
*Binderoboletus segoi*	BRG 41206	Guyana, Region 8 Potaro-Siparuni, Pakaraima Mountains	LC043078	LC043078	–	LC043079

### Phylogenetic analyses

For the combined ITS-nLSU-*TEF1-RPB1* dataset, we retrieved 170 sequences from INSD (Table 1). The sequences were carefully selected so that the dataset could represent all genera and subgenera in the subfamily *Leccinoideae*, which includes the genera *Chamonixia, Leccinellum, Leccinum, Octaviania, Rossbeevera* and *Turmalinea* (Orihara et al. 2016a). We selected *Spongispora temasekensis, Spongiforma thailandica, Borofutus dhakanus, Tylocinum griseolum, Binderoboletus segoi,* and *Retiboletus* spp. in subfamily *Leccinoideae* as outgroup taxa based on Henkel et al. (2016) and Wu et al. (2016, 2018). We only included specimens in our analysis whose nucleotide sequences covered more than 50% of the total length of the aligned, full ITS-nLSU-*TEF1-RPB1* dataset to reduce a negative effect caused by the lack of sequences in the dataset (i.e., no less than 1802 bp in length in the concatenated 4-gene dataset). Accordingly, we could not include sequences of *Ionosporus*, *Rhodactina*, *Pseudoaustroboletus* and two of the polyphyletic clades of *Leccinum* in the *Leccinoideae* previously shown in Kuo and Ortiz-Santana (2020); i.e. the *Leccinum talamancae* and *L. longicurvipes* lineages. Similarly, several species of *Octaviania*, including *O. asterosperma s. str.* and *O. arbucalensis*, which belong to subg. *Octaviania* (Yang et al. 2006; Vadthanarat et al. 2018), were not included in the analyses due to insufficient number of DNA loci available from INSD. Instead, we prepared an additional single-gene nLSU dataset that included as many *Octaviania* species as possible available from INSD, including the type species *O. asterosperma s. str.* (Table 1). The ML analyses were conducted with RAxML 8.2.10 (Stamatakis 2014) under the GTR + I + G model. The BioNJ analyses were conducted with SeaView version 4 (Gouy et al. 2010). Sequence alignment was performed with the online version of MAFFT version 7 (Katoh and Standley 2013) under default settings (i.e., the alignment algorithm is automatically selected from FFT-NS-1, FFT-NS-2, FFT-NS-i or L-INS-i). Subsequently, the sites with obvious alignment errors were manually adjusted in SeaView version 4. We referred to the results of the GBlocks option in SeaView (Castresana 2000) to exclude ambiguously aligned sites. Accordingly, the longest part of the insert within the ITS2 region found in all known species of *O.* subg. *Octaviania* (Orihara et al. 2012a) was excluded from our analyses. Prior to the multigene analyses, we compared the BioNJ tree topologies among the ITS, nLSU, *TEF1* and *RPB1* datasets to see if there were any topological conflicts among the gene trees. Sequences that caused considerable topological conflicts (BioNJ bootstrap values ≥75%; 1000 replicates) among the four single-locus phylogenies were excluded from the multilocus analyses. Accordingly, *RPB1* sequences of “*Octaviania tenuipes*” *nom. prov.* KPM-NC 27968 (INSD acc. no.: MT868858) and “*Octaviania potteri*” *nom. prov.* KPM-NC 17828 (MT868837 & MT868838), that are proposed as new taxa in this study were omitted from the combined multilocus dataset. We subsequently concatenated the four datasets for the multilocus analyses. The ITS rDNA region was partitioned by ITS1 + ITS2 and 5.8S, and the *TEF1* and *RPB1* regions were partitioned by codons and introns, and best-fit likelihood models were estimated for each partition and nLSU with MrModeltest 2.3 (Nylander 2004).

Bayesian analyses were conducted with MrBayes 3.2 (Ronquist and Huelsenbeck 2003). Nucleotide substitution models for maximum likelihood (ML) analyses were selected by the Akaike Information Criterion (AIC) in jModeltest2 (Darriba et al. 2012; Guindon and Gascuel 2003). The GTR + I + G model was applied to ITS1 + ITS2, nLSU, the second codon of *RPB1* and the first and third codons of *TEF1*; SYM + I for 5.8S rDNA; F81 + I for the second codon of *TEF1*; HKY + I for the first codon of *RPB1*; HKY + G for the third codon of *RPB1*; and HKY + I + G for the introns of *TEF1* and *RPB1*. Bayesian posterior probabilities (PP) were estimated by the Metropolis-coupled Markov chain Monte Carlo method (Geyer 1991). In the multi-gene (ITS + nLSU + *TEF1* + *RPB1*) analysis, two parallel runs were conducted with one cold and seven heated chains each for 4 M generations. The parameter for temperature of the seven heated chains in both runs was set to 0.10. The 0.10 heating scheme was used instead of the default 0.20 setting, because in previous phylogenetic studies on the *Leccinoideae*, the Markov chains with the 0.10 heating setting converged more smoothly and were less likely to become trapped at local optima (Orihara et al. 2016a; Orihara and Smith 2017). Trees were saved to a file every 1000th generation. We determined that the two runs reached convergence when the average standard deviation of split frequencies (ASDSF) was continuously lower than 0.01. The ASDSF was monitored every 5000 generations. We also verified the convergence by checking that the effective sample size (ESS) of each resulting statistic was sufficiently large (> 200). Trees obtained before reaching convergence were discarded as the burn-in, and the remaining trees were used to calculate a 50% majority consensus topology and to determine PP values for individual branches.

Maximum likelihood (ML) analyses were conducted with RAxML 8.2.10. The same partitioned datasets as those for the Bayesian analyses were used so that different α-shape parameters, GTR rates, and empirical base frequencies could be assigned to each partition. The best-fit ML tree was estimated under the GTRCAT+I model. The rapid bootstrap (BS) analysis was implemented with 1000 replicates.

The single-gene nLSU phylogenies that included all the representative species of *Octaviania* available from INSD were estimated using the ML and BioNJ methods. The ML analysis was conducted using RAxML 8.2.10, setting the substitution model to GTRCAT+I and the number of rapid BS replicates to 1000. The BioNJ analysis was done by SeaView version 4 with the number of BS replicates set to 1000.

To compare tree topologies and examine precise phylogenetic placement of our three target taxa in *Octaviania* subg. *Octaviania*, we further inferred ML gene trees from individual ITS, nLSU, *RPB1*, and *TEF1* datasets of the subgenus using RAxML 8.2.10. The datasets were partitioned by genes for ITS (i.e., ITS1 + ITS2 and 5.8) and by codons for *RPB1* and *TEF1*. The best-fit ML tree was estimated under the GTRCAT+I model. The rapid BS analysis was implemented with 1000 replicates.

Since the comparison of the four gene trees of *Octaviania* subg. *Octaviania* detected several heterogenous sequences in the *RPB1* and *TEF1* regions, we further conducted phylogenetic network analysis based on a smaller multilocus dataset to find the traces of reticulate evolution among infrageneric taxa in the subgenus *Octaviania*. The dataset for this analysis included 1–2 representative specimens for each species of the subgenus *Octaviania.* We selected specimens for which molecular data were available from all four DNA regions (i.e. ITS, nLSU, *RPB1* and *TEF1*). The *RPB1* sequences of “*Octaviania tenuipes*” *nom. prov.* (MT868858 [KPM-NC 27968]) and “*Octaviania potteri*” *nom. prov.* (seq1: MT868837 [KPM-NC 17828; *RH1181*]), which were omitted in the multi-gene Bayesian and ML analyses discussed above, were included in the combined dataset for this analysis. The analysis was executed with SplitsTree 4 (Huson and Bryant 2006). Networks were constructed by the NeighborNet method using the “distance estimation to uncorrected P value” setting. The resultant networks were displayed with the EqualAngle algorithm (Dress and Huson 2004). Bootstrap analysis was then conducted with 1000 replicates.

## RESULTS

### Morphological evaluation of the north American species of *Octaviania* subgenus *Octaviania*

The phylogenetic analyses in Orihara et al. (2012a) explicitly showed that three specimens of *Octaviania* (KPM-NC 17827, KPM-NC 17828 and OSC 13925) from eastern North America (i.e. Iowa, Minnesota and Florida) formed a distinct clade within *Octaviania* subg. *Octaviania* but provided no taxonomic treatment of the unidentified taxon. We critically examined the morphology and habitat of the taxon and we compared it with the previously published literature on North American *Octaviania* species.

The overall macro-morphology, peridial structure and the basidiospore and basidia dimensions matched the original description of *Octaviania asterosperma* var. *potteri* Singer and Smith (Singer and Smith 1960), which was reported from Michigan, USA (see description of *O. potteri* below). We studied the holotype of *O. asterosperma* var. *potteri* (MICH 12376 [*Potter 8898*]) in MICH, which was well-preserved, but the cells of the peridium were collapsed. The basidiospore morphology matched that of the three North American specimens of *Octaviania* sp. (Table 2). We therefore identify the North American *Octaviania* species as *O. asterosperma* var. *potteri*. Below we propose a new status as *O. potteri* stat. nov.

**Table 2:** Comparison of basidiospore dimentions between holotype of *O. asterosperma* var. *potteri* and a recently collected North American specimen (KPM-NC 17827)

	Holotype (MICH 0001237) from Michigan, USA	KPM-NC 17827 (RH30) from Iowa, USA
Basidiospore size [average (*n* = 30)]	9–14 × 8.8–13.2 μm [11.3 × 10.5 μm]	9.6–13.9 × 7.6–12.7 μm [11.2 × 9.7 μm]

### Phylogenetic placement of new taxa inferred from the multilocus phylogeny

The multilocus dataset comprised of ITS and LSU nrDNA, *TEF1* and *RPB1* sequences of the *Leccinoideae* consisted of 94 specimens and 3603 aligned nucleotide positions. The Bayesian inference reached convergence after ca. 1.38 M generations. Accordingly, we discarded the first 1400 trees in each parallel run, and the remaining 2601 trees in each run were summarized to approximate Bayesian posterior probabilities (PPs). ESS of all the model parameters were sufficiently large (> 200). The total arithmetic and harmonic mean of estimated marginal log likelihoods (lnL) for runs were − 27576.51 and − 27653.13, respectively. In the RAxML analysis, the final ML optimization of log likelihood was − 27424.085894. The overall topologies between the Bayesian and ML trees were nearly identical.

**Fig. 1:**
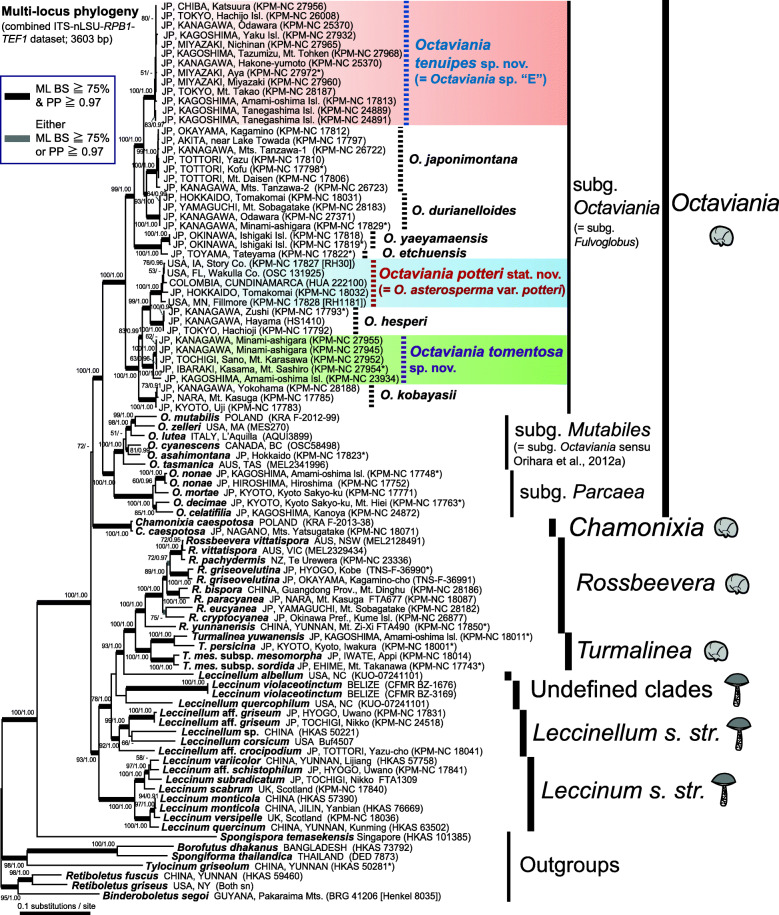
Maximum likelihood (ML) tree of the *Leccinoideae* inferred from the multilocus (ITS and LSU nrDNA, *RPB1* and *TEF1*) dataset. Branches supported by both BS ≥ 75% and PP ≥ 0.97 and are depicted as thickened black lines. Branches supported by either BS ≥ 75% or PP ≥ 0.97 are shown as thickened gray lines. Statistical values below BS < 50% or PP < 0.90 are not shown. Holotype materials are designated with asterisks (*). Taxa outside of the core clade of *Leccinoideae* are used as outgroups

The resulting phylogenetic trees (Fig. 1) robustly recovered the known generic relationships within the *Leccinoideae*, some with higher statistical support than in previous studies (e.g., /*Spongiforma-Borofutus-Tylocinum* clade; Wu et al. 2016, /*Leccinum-Leccinellum-Turmalinea-Rossbeevera* clade: Wu et al. 2018; Orihara et al. 2016a; Kuo and Ortiz-Santana 2020). *Octaviania* sp. “E” (i.e. *O. tenuipes* sp. nov.) from Japan and *O. asterosperma* var. *potteri* from North America (i.e. *O. potteri* stat. nov.) were placed within *Octaviania* subg. *Octaviania*, as shown by Orihara et al. (2012a). A previously unknown species-level clade (*O. tomentosa* sp. nov.) was also placed within *Octaviania* subg. *Octaviania*.

Specimens of *O. tenuipes* sp. nov. exhibited minimal infraspecific genetic divergence. In contrast, both *O. potteri* stat. nov. and *O. tomentosa* sp. nov. showed considerable genetic divergence among specimens. In the *O. tomentosa* clade, a specimen from Amami-oshima Island in the Ryukyu island chain, was genetically divergent from the other specimens from mainland Japan. In the *O. potteri* stat. nov. clade, the geographically isolated specimens from Hokkaido, Japan (KPM-NC 18032) and Colombia (HUA 222100) were nested among the North American specimens.

Although the generic type species, *O. asterosperma,* was not included in the multilocus phylogenies, the nLSU gene tree indicate that *O. potteri* stat. nov. is genetically distant from *O. asterosperma* var. *asterosperma* and it should be treated as a distinct taxon (Fig. S1; the lnL of the ML tree = − 3239.444877).

### Comparison of single-gene tree topologies within *Octaviania* subgenus *Octaviania*

**Fig. 2:**
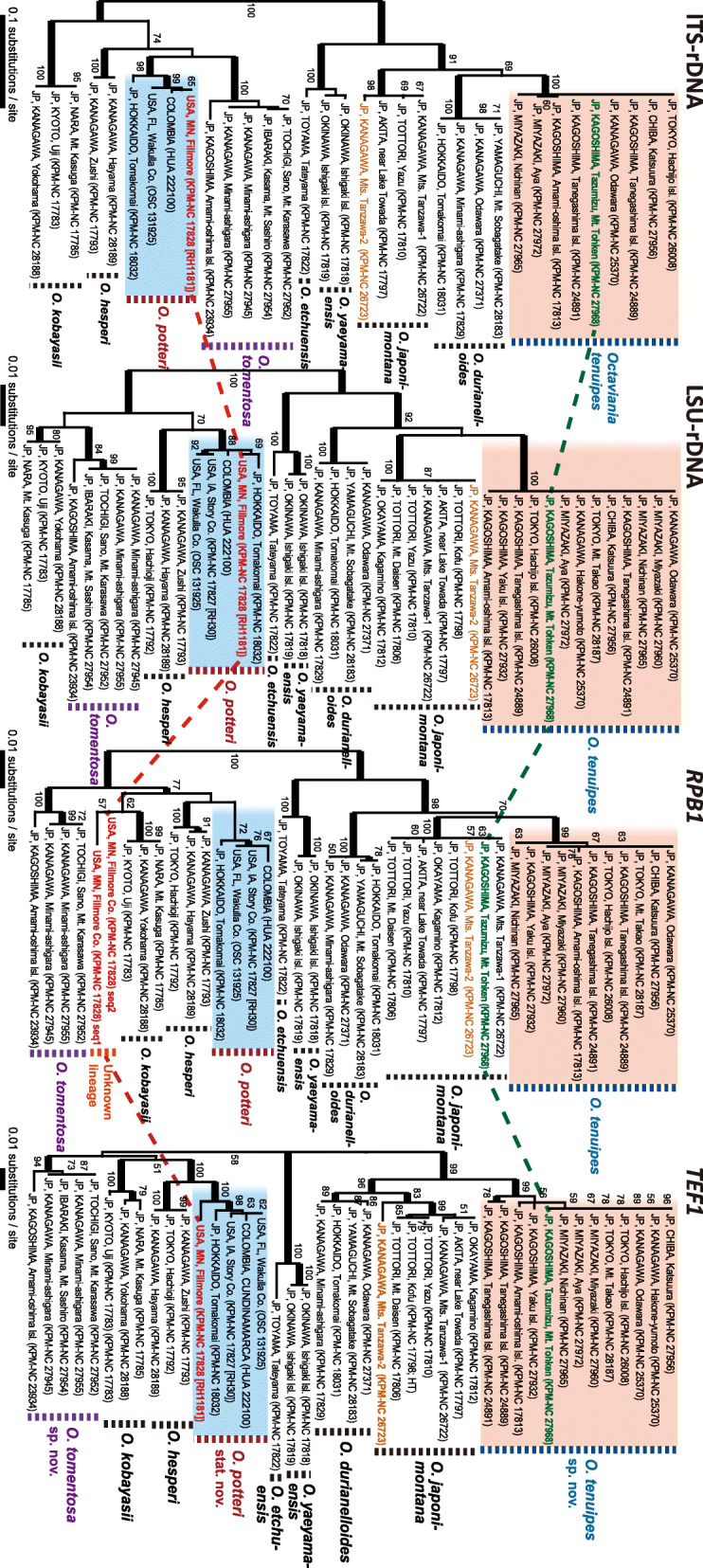
Comparison of four ML trees of *O.* subg. *Octaviania* based on ITS and LSU nrDNA, *RPB1* and *TEF1* datasets. Branches supported by BS ≥ 75% are depicted as thickened black lines. BS value below 50% are not shown. Specimens that have heterogeneous *RPB1* or *TEF1* sequences are depicted in coloured fonts. *Octaviania potteri* KPM-NC 17828 (RH1181) specimen had two highly heterogenous *RPB1* sequences (i.e. “seq1” and “seq2”) as shown in red font

The four ML gene trees based on ITS nrDNA (ITS1-5.8S-ITS2), LSU nrDNA, *RPB1* and *TEF1* datasets were estimated with the final ML optimization of lnL of − 1806.362004, − 1975.613722, − 2139.994105 and − 2417.247141, respectively (Fig. 2). All of the species-level clades in subg. *Octaviania* except *O. potteri* stat. nov. were recovered in each tree with high bootstrap values.

Interestingly, one *O. tenuipes* specimen collected from a *Castanopsis sieboldii* forest in Mt Tohken, Kagoshima Prefecture, Japan (KPM-NC 27968) had an identical *RPB1* sequence to *O. japonimontana*, which was inferred to be sister to *O. tenuipes* in the *RPB1* tree with moderate BS support. This result was confirmed by sequencing the *RPB1* region of the specimen twice using different primer pairs. Furthermore, one *O. potteri* specimen from Minnesota, USA (*RH1181*; KPM-NC 17828) had at least two heterogeneous *RPB1* sequences, one of which was apparently derived from *O. potteri* but another was remarkably divergent from the other *O. potteri* sequences. The ML phylogeny showed that the divergent sequence from the *RH1181* specimen forms its own clade and is an unknown species-level lineage that is sister to *O. kobayasii* (Fig. 2). It should also be noted that the *TEF1* sequence of a *O. japonimontana* specimen from the Tanzawa mountains, Kanagawa Prefecture (KPM-NC 27623; Fig. 4g) was remarkably divergent from the other *O. japonimontana* sequences (i.e., the *TEF1* identity between the two specimens from the Tanzawa moutains [KPM-NC 27622, 27623] was 98.96% [1051/1060 bp]) despite the high sequence homogeneity of *O. japonimontana* in the other three regions. For comparison, the *TEF1* sequence identity between *O. tenuipes* (INSD, acc. no. MT874813) and *O. durianelloides* (MT874817) was 98.65% (1094/1109 bp).

### Network analysis based on the multilocus dataset of subgenus *Octaviania*

**Fig. 3:**
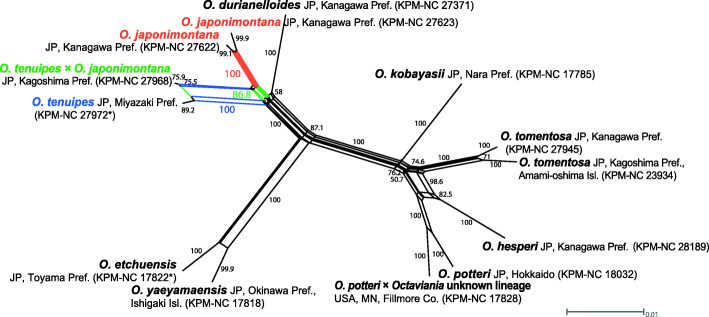
Phylogenetic network of the combined multilocus (ITS and LSU nrDNA, *RPB1* and *TEF1*) dataset of *O.* subg. *Octaviania* constructed by the NeighborNet method and displayed with the EqualAngle algorithm (Dress and Huson 2004). BS values below 50% are not shown. Holotype materials are designated with asterisks (*)

The network analysis of the multilocus dataset supported the *O. tenuipes* specimen with the *RPB1* sequence of *O. japonimontana* (KPM-NC 27968; Fig. 2) as an intermediate lineage between *O. tenuipes* and *O. japonimontana*, showing a high degree of reticulation in the tree (Fig. 3). This relationship was supported with high bootstrap values (86.8–100%). On the other hand, no other clear reticulations suggest recent hybridization among species in subg. *Octaviania*.

## TAXONOMY

Based on our morphological studies and phylogenetic results (Fig. 1), we describe two new species, *O. tenuipes* and *O. tomentosa*, from Japan. The multilocus phylogenetic analyses also strongly support *O. potteri* stat. nov. as sister to *O. hesperi* in *O.* subg. *Octaviania* (Fig. 1). Furthermore, our nLSU gene tree shows that *O. potteri* stat. nov. is phylogenetically distant from *O. asterosperma* var. *asterosperma* (Fig. S1). We conclude that the taxon previously considered as *O. asterosperma* var. *potteri* is a distinct species from *O. asterosperma s. str.*, and we propose a new status, *Octaviania potteri*, for thistaxon.

We are aware of the work by Kuo and Ortiz-Santana (2020) that proposed the synonymy of the sequestrate genera *Octaviania, Chamonixia, Turmalinea,* and *Rossbeevera* within a broadly circumscribed genus *Leccinum s.lat.* While their phylogenetic analyses resolved monophyletic clades for all of these sequestrate genera, several other major clades of epigeous *Leccinoideae* were otherwise poorly resolved. Based on the lack of resolution in their phylogenetic trees and the uncertainty that remains about the evolutionary relationships within the *Leccinoideae*, we opt to retain the sequestrate genus names *Octaviania, Chamonixia, Rossbeevera,* and *Turmalinea*. We acknowledge that these sequestrate taxa belong to the *Leccinoideae* but feel that synonymy with *Leccinum* is premature, results in the loss of information, and does little to clarify the taxonomy and phylogeny of this group. In our multilocus phylogeny, some relationships within *Leccinoideae* clades that were unresolved in Kuo and Ortiz-Santana (2020) were resolved with high statistical support (i.e., ML BS ≥ 75% and PP ≥ 0.97; Fig. 1). For example, phylogenetic placement of the core clade of *Leccinum* (i.e. *Leccinum s.str.*) was not resolved in Kuo and Ortiz-Santana (2020), whereas our phylogeny supported *Leccinum s. str.* as sister to the clade comprised of *Leccinellum s. str.*, two undefined leccinoid clades, *Rossbeevera,* and *Turmalinea* (Fig. 1). Our phylogeny also supported monophyly of the *Leccinellum s. str.* clade, which was not strongly supported in Kuo and Ortiz-Santana (2020). However, we could not include two independent clades, *Leccinum talamancae* and *L. longicurvipes*, whose phylogenetic positions within the *Leccinoideae* remained uncertain. Our results suggest that we need to further address the phylogeny and systematics of *Leccinoideae* before lumping together all the well-defined epigeous and sequestrate genera into one large and broadly circumscribed genus. We will wait to determine a final taxonomic scheme for the sequestrate genera until a more highly resolved phylogeny becomes available that provides appropriate insight into this group.

***Octaviania tenuipes*** Orihara, **sp. nov.**

MycoBank MB 836874

(Fig. 4a–f)

**Fig. 4:**
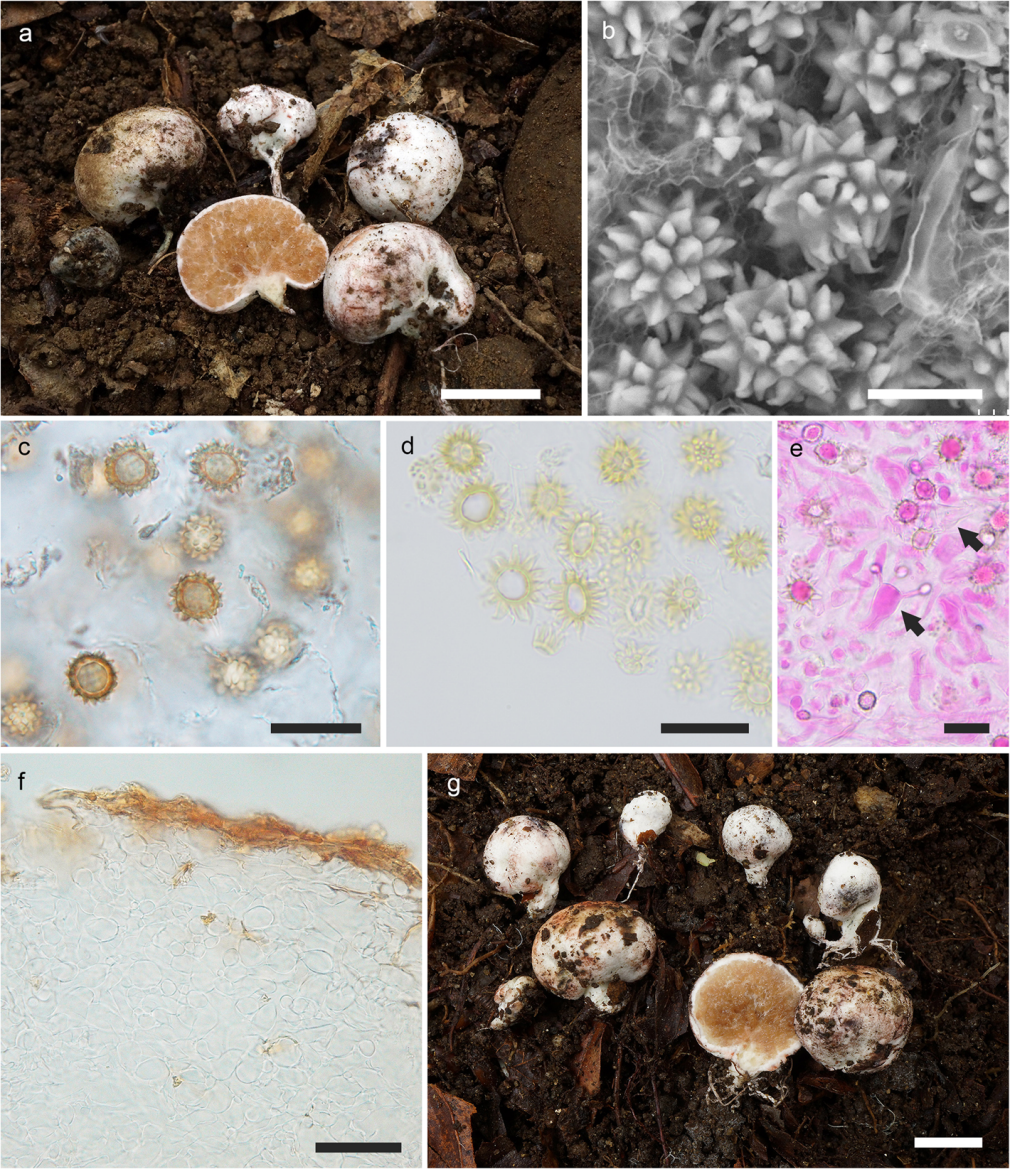
**a–f** *Octaviania tenuipes*. **a** Basidiomata (holotype [KPM-NC 27972]). **b** Basidiospores under SEM (holotype). **c** Basidiospores mounted in water (KPM-NC 27957). **d** Basidiospores with elongated ornamentation mounted in lactic acid after pre-soaking in 3% KOH (holotype). **e** Basidia (arrows) and basidiospores mounted in 3% KOH after staining with 1% phloxine (holotype). **f** Peridium (holotype). **g** Basidiomata of *O. japonimontana* (KPM-NC 27623). Topological comparison among gene trees reveals that this specimen has a remarkably divergent *TEF1* sequence from those of other *O. japonimontana* specimens (Fig. 2). Scale bars: **a**, **g** = 1 cm, **b** = 10 μm, **c–e** = 20 μm, **f** = 50 μm

*Synonym*: *Octaviania* sp. “E” Orihara et al., *Persoonia* **28**: 104 (2012); as “nom. prov.”.

*Etymology*: Latin, *tenuis* (slender) and *pes* (foot, stipe), referring to the slender stipe (sterile base) of the species, which is unique in the genus.

*Diagnosis*: Morphologically similar to some species in *Octaviania* subg. *Octaviania*, but is distinguished by the combination of the following characteristics: soft, whitish basidiomata with a more or less developed stipe sometimes exceeding 10 mm long at the base; a thin peridium that is mostly less than 0.5 mm thick; basidiospores 9.3–(9.5–)13.3(− 15) × (8.4–)8.5–11.3(− 11.5) μm with acute pyramidal spines that have one simple cavity inside.

*Type*: **Japan**: *Miyazaki Pref*.: Aya Town, under *Castanopsis sieboldii*, 26 Nov. 2012, *T. Orihara & S. Kurogi* (KPM-NC 27972 – holotype).

*Description*: *Basidiomata* sequestrate, to 21 mm diam, soft, depressed-globose or reniform; surface smooth or slightly floccose, white at first, becoming dirty white to light yellowish brown at maturity, turning immediately wine red when immature specimens are touched, immediately blue when mature specimens are touched, gradually oxidizing further to black, with a more or less developed stipe that sometimes exceeds 10 mm long, becoming conspicuously slender at the bottom. *Peridium* mostly less than 0.5 mm thick. *Gleba* whitish in youth, then becoming brown and finally blackish brown, somewhat watery, composed of darker-coloured locules filled with basidiospores and whitish mycelial veins. *Stipe* (sterile base) well developed compared to other typical species of the genus, often with some subhyaline spots inside, occasionally turning red (in immature basidiomata) or blue (in mature basidiomata) when cut. *Odour* fragrant.

*Basidiospores* 9.3–(9.5–)13.3(− 15) × (8.4–)8.5–11.3(− 11.5) μm, mean 11.3 × 9.9 μm (SD: 1.00 [length], 0.67 [width]), subglobose to broadly ellipsoid (*Q* = 1–1.42, *Q*_*m*_ = 1.14), light yellowish brown to brown, covered with coarse, acute, pyramidal spines 1.1–3.3 μm high and 1–4.5 μm wide with a single, simple cavity inside; spore walls 1–1.6 μm thick. *Basidia* 21–32 × 8–14 μm, mean 26 × 11.1 μm, clavate, hyaline, 2-, 3- or 4-spored. *Hymenium* present but poorly developed, comprised of basidia and basidioles. *Subhymenium* absent; basidia connected to branched filamentous hyphae directly extending from trama. *Trama* hyaline, of subparallel to loosely interwoven, non-inflated, thin-walled (to 0.8 μm) filamentous hyphae 2–9 μm broad. *Peridium* mostly 100–400 μm, sometimes to 650 μm thick, of densely interwoven, often inflated filamentous hyphae 2–17 μm broad when immature, gradually inflated with age up to 40 μm diam, becoming pseudoparenchymatous cells at maturity; walls 0.5–1.2 μm thick; outermost hyphae pigmented brown to fuscous, somewhat narrower, up to 10 μm broad, but not forming a distinct layer. *Stipe* (sterile base) of compactly interwoven, hyaline, thin-walled, inflated hyphae 3–22 μm broad, partially intermingled with large, irregular-shaped, pseudoparenchymatous cells to 60 μm in diam, walls 0.5–1.3 μm thick. *Clamp connections* absent in all tissues.

*Habitat, distribution, and seasonality*: Hypogeous or subhypogeous under evergreen *Fagaceae*; widely distributed throughout Japan; spring to early summer and autumn to early winter.

*Other specimens examined*: **Japan:** *Tokyo Met.*, Hachioji City, Mt Takao, 7 Sep. 2015, *M. Nakajima* (KPM-NC 28187); Hachijo Island, Hachijo Town, along Boh-ei Rd., under *Castanopsis sieboldii*, 31 Oct. 2003, *H. Sasaki 257* (KPM-NC 28191); ibid, *H. Sasaki 261* (KPM-NC 28192); Hachijo Island, Hachijo Town, Mitsune, Kamogawa Forestry Rd., under *C. sieboldii*, 15 Jul. 2015, *A. Hosono* (KPM-NC 27958); Hachijo Island, Hachijo Town, Ohkagoh, under *C. sieboldii*, 26 Apr. 2017, *T. Orihara* (KPM-NC 26008); ibid, 29 Jun. 2003, *H. Sasaki 157* (KPM-NC 28190); ibid, 2 Jul. 2005, *H. Sasaki 567* (KPM-NC 28193); *Chiba Pref.*, Katsuura City, Okitsu, under *Lithocarpus edulis*, 8 May 2016, *T. Kasuya* (KPM-NC 27956); *Kanagawa Pref.*, Hakone Town, Hakone-yumoto, Soh-un Park, under *C. sieboldii*, 2 Oct. 2016, *T. Orihara* (KPM-NC 27957); Odawara City, Iryuda, near Myoriki-ji Shrine, under *C. sieboldii*, 1 Dec. 2016, *M. Nakajima* (KPM-NC 25370); *Miyazaki Pref.*, Miyazaki City, Tano-cho-otsu, Tano Forest Science Station, Miyazaki Univ., under *C. cuspidata* and *Quercus glauca*, 22 Nov. 2012, *T. Orihara* (KPM-NC 27960); Nichinan City, Inohae Valley, 23 Nov. 2012, *T. Orihara* (KPM-NC 27965); ibid, under *Q. gilva* and *Q. salicina* (KPM-NC 27964); *Kagoshima Pref.*, Tarumizu City, Mt. Tohken, under *C. sieboldii*, 24 Nov. 2012, *T. Orihara* (KPM-NC 27968); Kimotsuki-gun Minamiosumi Town (the former Sata Town), Nishikata, under *C. sieboldii*, 30 Nov. 2003, *H. Sasaki 306* (KPM-NC 28405); ibid, *H. Sasaki 308* (KPM-NC 28406); ibid, *H. Sasaki 309* (KPM-NC 28407); ibid, *H. Sasaki 310* (KPM-NC 28408); ibid, *H. Sasaki 311* (KPM-NC 28409); ibid, *H. Sasaki 312* (KPM-NC 28410); Kimotsuki-gun Minamiosumi Town (the former Sata Town), near Kaitaku-iriguchi bus stop, under *C. sieboldii* and *Q. glauca*, *H. Sasaki 317* (KPM-NC 28411); Tanegashima Isl., Nishino-omote City, Anjoh, Ohno Forestry Rd., along Ohkawada River, under *C. sieboldii* and *Q. glauca*, 8 Dec. 2015, *T. Orihara* (KPM-NC 24889); Nishino-omote City, Furuta under *Lithocarpus edulis*, 28 Nov. 2003, *H. Sasaki 301* (KPM-NC 28404); ibid, under *C. sieboldii*, 8 Dec. 2015, *T. Orihara* (KPM-NC 24891); Tanegashima Isl., Minamitane Town, Nakanoshita, near Shimonakahachiman Shrine, under *Castanopsis sieboldii, Quercus phillyraeoides* and *Lithocarpus edulis*, 28 Nov. 2003, *H. Sasaki 294* (KPM-NC 28401); ibid, *H. Sasaki 295* (KPM-NC 28402); Tanegashima Isl., Nakatane Town, Masuda, near Tanegashima Airport, 28 Nov. 2003, *H. Sasaki 298* (KPM-NC 28403); Amami-oshima Isl., Yamato-son, north-eastern foot of Mt Yuwan, umder *C. sieboldii* subsp. *lutchuensis*, 17 Nov. 2007, *T. Orihara* (KPM-NC 17813).

*Remarks*: Orihara et al. (2012a) tentatively described *O. tenuipes* as “*Octaviania* sp. E” because, at that time only one collection of an immature basidiome had been examined and the morphology of the new species was not sufficiently known. This species has now been recorded from subtropical to temperate regions in Japan, associated with *Castanopsis, Lithocarpus* and evergreen *Quercus* [= *Cyclobalanopsis*] tree species. Morphologically, *O. tenuipes* tends to have a rather slender and well-developed stipe compared to the other species of *Octaviania*. *Octaviania japonimontana*, which is phylogenetically close to *O. tenuipes*, is somewhat similar morphologically, but *O. japonimontana* occurs in deciduous *Fagaceae* forests (with *Q. crispula* and *Fagus* spp.) and tends to have basidiomes with thicker peridia and a more rubbery texture. However, these differences are sometimes inconspicuous so molecular methods are sometimes necessary to confirm the species identification. Another closely related species, *O. durianelloides*, also resembles *O. tenuipes* when the basidiomata are immature. However, at maturity the basidiomes of *O. durianelloides* have conspicuous brown scales or warts on the surface, which is unique in the genus.

***Octaviania tomentosa*** Orihara, **sp. nov.**

MycoBank MB 836875

(Fig. 5)

**Fig. 5:**
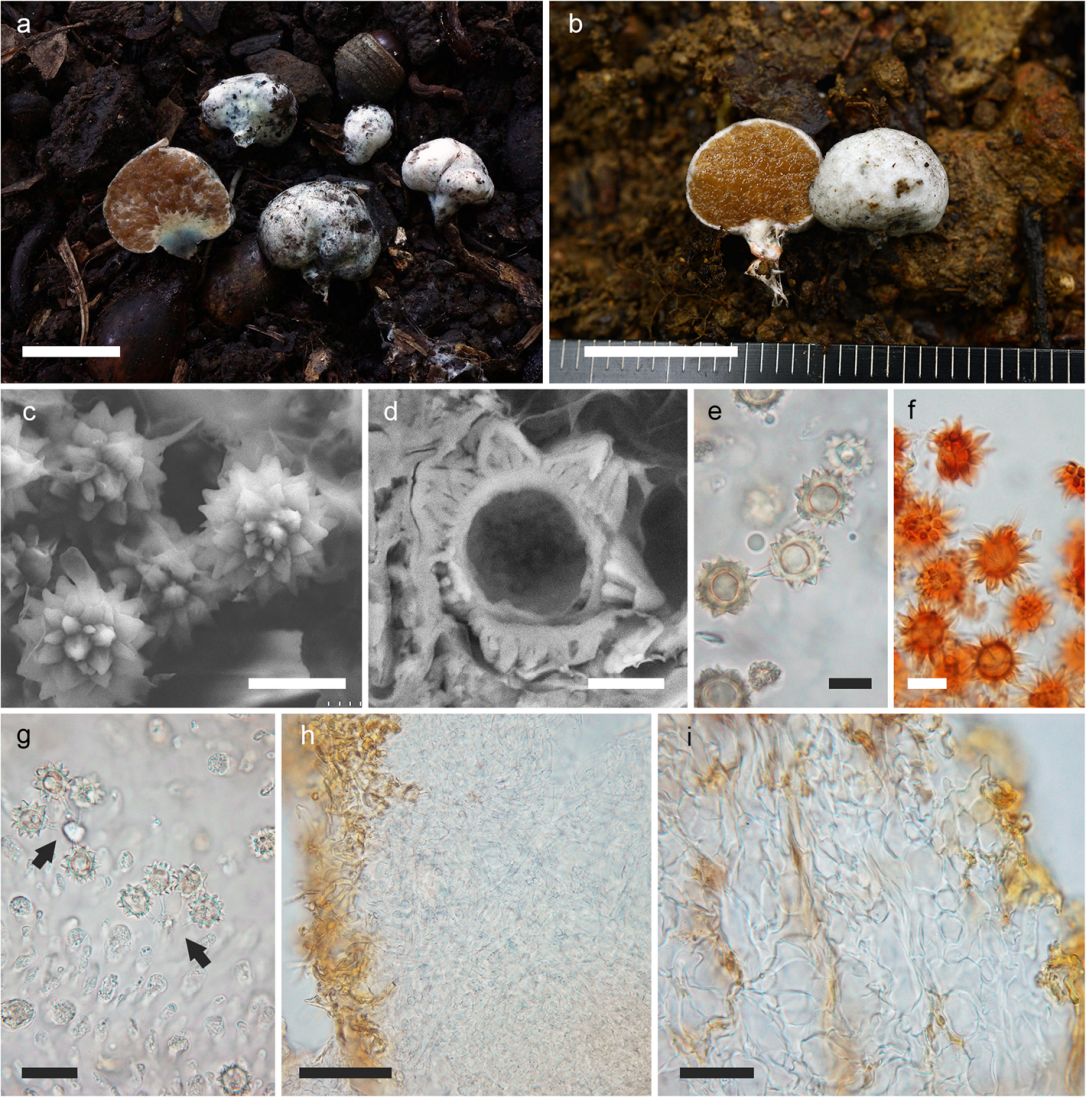
*Octaviania tomentosa*. **a–b** Basidiomata (**a** holotype from central Honshu, Japan [KPM-NC 27954]; **b** specimen from Amami-oshima Island, Japan [KPM-NC 23934]). **c** Basidiospores under SEM (holotype). **d** section of a basidiospore showing multiple slit-like cavities inside the spiny ornaments. **e** Basidiospores mounted in water (KPM-NC 27945). **f** Dextrinoid basidiospores with elongated ornamentation mounted in lactic acid after pre-soaking in Melzer’s reagent (KPM-NC 23934). **g** Basidiospores connected to 4-spored basidia (arrows; KPM-NC 27955). **h** Peridium of an immature basidiome (KPM-NC 27955). **i** Peridium of a mature basidiome (KPM-NC 27953). Scale bars: **a**–**b** = 1 cm, **c**, **e**–**f** = 10 μm, **d** = 5 μm, **g**, **i** = 20 μm, **h** = 50 μm

*Etymology*: Latin, *tomentosa* (felty or cottony), referring to the tomentose surface of the basidiomata.

*Diagnosis*: Distinguished from other *Octaviania* species in the combination of the following characteristics: small, soft, felty to tomentose, white to dirty white basidiomata to 15 mm diam; peridium usually very thin (mostly 70–250 μm thick), composed of filamentous hyphae and isodiametric cells with thin cell-walls (to 0.8 μm thick); and basidiospores 10.2–(11–)14.4(− 15) × 8.6–(8.8–)12.6(− 13.2) μm, with acute or sometimes curled, pyramidal spines that have a few slit-like cavities inside.

*Type*: **Japan:** *Ibaraki Pref.*: Kasama City, Mt Sashiro, under *Quercus myrsinifolia*, 9 Sep. 2018, *M. Ohmae & T. Orihara* (KPM-NC 27954 – holotype).

*Description*: *Basidiomata* sequestrate, to 15 mm diam, soft, subglobose, depressed-globose or reniform; surface felty to tomentose, white to dirty white, not becoming yellowish or brownish with age, turning blue or bright red when touched or injured; immature basidiomata tending to turn red rather than blue where touched, after exposure gradually turning black; stipe short, not exceeding 3 mm long, with white rhizomorphs. Peridium usually less than 0.25 mm, occasionally up to 0.4 mm, context white, showing the same pattern of discoloration as the peridial surface. *Gleba* beige in youth, becoming brown at maturity, somewhat watery, particularly in young basidiomata, composed of locules filled with yellowish brown to brown basidiospores and whitish mycelial veins, typical of the genus. *Stipe* (sterile base) context white, sometimes with some subhyaline spots inside. *Odour* fragrant, fruity at maturity.

*Basidiospores* 10.2–(11–)14.4(− 15) × 8.6–(8.8–)12.6(− 13.2) μm, mean 12.3 × 10.6 μm (SD: 1.01 [length], 0.96 [width]), subglobose to broadly ellipsoid (*Q* = 1.04–1.34, *Q*_*m*_ = 1.16), light yellowish brown to ochraceous brown, covered with coarse, acute, sometimes curled, large pyramidal spines 1.6–3.3 μm high and 1.5–4.8 μm wide with a few slit-like cavities inside; spore walls 1.3–3 μm thick, with a long pedicel 6–15.5 × 1.5–2.2 μm at the base. *Basidia* 26–39 × 9–14 μm, mean 32.1 × 11.1 μm, clavate, colourless, 4-, 2- or more rarely 3-spored. *Subhymenium* not well developed. Basidia and basidioles randomly extending from hyphae in tramal plates. *Tramal plates* 15–80 μm thick, of parallel, colourless, thin-walled (to 0.6 μm) filamentous hyphae 2.5–7 μm broad. *Peridium* usually 70–250 μm thick, context to 180 μm thick, colourless, of interwoven, septate, thin-walled (to 0.6 μm) filamentous hyphae approximately 3–8 μm broad when immature, cells becoming swollen and isodiametric (to 25 μm diam) so that peridial tissue is pseudoparenchyma by maturity; the mature cell walls up to 0.8 μm thick; peridiopellis thin, to 100 μm across, pigmented yellow-brown, surface turf-like but fragile and easily crushed, of interwoven filamentous hyphae or inflated cells almost the same size as those of inner context (to 25 μm diam). *Stipe* (sterile base) of compact, interwoven, partially isodiametric, thin-walled (to 0.8 μm), hyaline hyphae 4–20 μm broad. *Clamp connections* absent in all tissues.

*Habitat, distribution and seasonality*: Hypogeous or subhypogeous under evergreen *Fagaceae*, found on Amami-oshima Island in the Ryukyu island chain and in eastern Honshu (Kanto region), Japan; summer to autumn.

*Other specimens examined*: **JAPAN:** *Kanagawa Prefecture*, Minami-ashigara City, Uchiyama, under *Quercus myrsinifolia*, 2 Nov. 2014, *H. Yamashita* (KPM-NC 25092); ibid, 4 July 2016, *T. Orihara* (KPM-NC 27945); ibid, 3 Sep. 2017, *T. Orihara* (KPM-NC 27955); ibid, 24 Sep. 2018, *T. Orihara*, KPM-NC 27946; ibid, 19 Jul. 2020, *Y. Kaneko & T. Orihara* (KPM-NC 28415); *Ibaraki Pref.*, Kasama City, Mt. Sashiro, under *Q. myrsinifolia*, 22 Jul. 2017, *M. Ohmae* (KPM-NC 27953); *Tochigi Pref.*, Sano City, Mt. Karasawa, under *Castanopsis sieboldii*, 24 Jul. 2016, *M. Ohmae* (KPM-NC 27952); ibid, 20 Jul. 2018, *M. Ohmae* (KPM-NC 27947); *Shizuoka Pref.*, Suntoh District, Oyama Town, Ashigara Pass, 12 Jul. 2020, *Y. Kaneko* (KPM-NC 28416); ibid, 19 Jul. 2020, *Y. Kaneko* (KPM-NC 28412); ibid, *Y. Kaneko & T. Orihara* (KPM-NC 28413); ibid, *Y. Kaneko* (KPM-NC 28414); *Kagoshima Pref.*, Amami-oshima Isl., Uken-son, Yuwan, umder *C. sieboldii* subsp. *lutchuensis*, 29 Jun. 2014, *T. Orihara* (KPM-NC 23934).

*Remarks*: This rare species has only been found in four sites in and around the Kanto region in Honshu and from one site in Amami-oshima Island in the Ryukyu island chain despite extensive long-term collecting of *Octaviania* spp. throughout Japan. These two disjunct areas are about 1200 km apart and the climate and vegetation are also quite different between the two areas (temperate evergreen forests on mainland Japan vs. subtropical forests in the Ryukyu Islands). The multilocus tree (Fig. 1) as well as the single-gene trees (Fig. 2) clearly show generic divergence between the two disjunct lineages. The specimen from Amami-oshima Island had a thicker peridium than the specimens from the Kanto region (ca. 150–400 μm thick in the Amami-oshima specimen vs. 70–250 um thick in specimens from Kanto). However, we treat these two lineages as infraspecific variation because of the lesser degree of genetic divergence compared to other species-level divergence in both the species tree and individual gene trees (Figs. 1 and 2). For instance, the sequence similarity of nLSU between the Amami-oshima specimen and the holotype from Honshu is 99.15% (935 bp / 943 bp), whereas the nLSU similarity between holotypes of *O. yaeyamaensis* and *O. etchuensis*, which are genetically the least divergent species within subgenus *Octaviania*, is 98.31% (875 bp / 890 bp). In addition, we cannot infer the potential mating incompatibility between these geographically isolated, but uncultured specimens.

*Octaviania tomentosa* morphologically resembles *O. hesperi* in the relatively small, whitish basidiomes. However, *O. hesperi* is distinguished from *O. tomentosa* by its slightly larger basidiospores with lower *Q* values (10–15.6(− 18.2) × 9.4–(9.9–)14.8(− 17.8) μm, mean 12.8 × 12.1 μm; *Q* = 0.96–1.15). *Octaviania hesperi* also has larger pyramidal spore ornamentation (2–(2.1–)3.6(− 4) × 1.3–(1.7–)5.4(− 5.7) μm) with multiple, irregularly shaped slits inside (Orihara et al. 2012a). The felty to tomentose surface of the *O. tomentosa* basidiomes is also a distinguishing character that is absent among any of the phylogenetically related species in *O.* subg. *Octaviania*.

***Octaviania potteri*** (Singer & A.H. Sm.) Orihara, Healy, M.E. Sm., **stat. nov.**

MycoBank MB 836876

(Fig. 6)

**Fig. 6:**
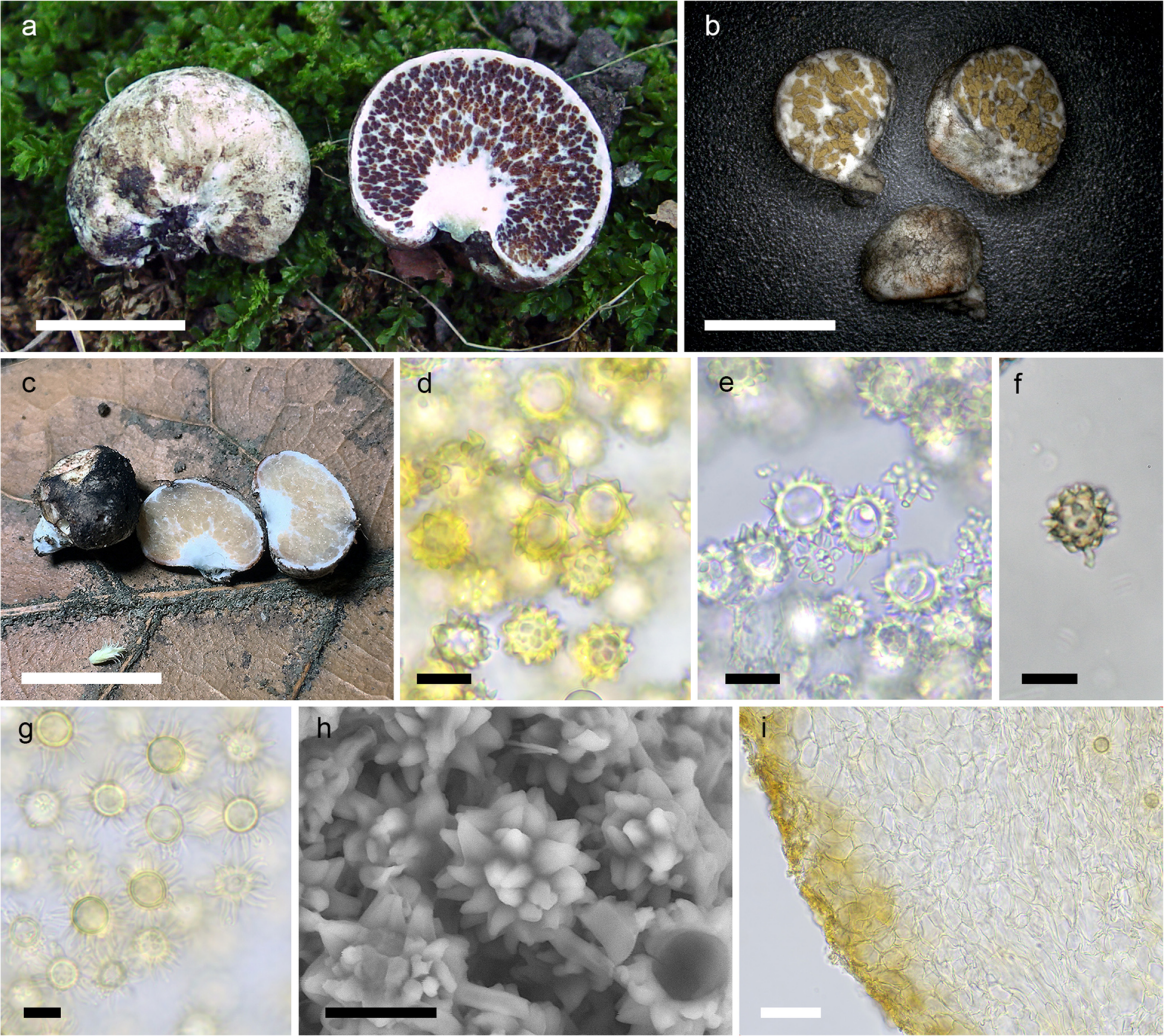
*Octaviania potteri*. **a–c** Basidiomata (**a** FH 00284311 [*RH30*; duplicate: KPM-NC 17827] from Iowa, USA; **b** KPM-NC 18032 from Hokkaido, Japan; **c** HUA 222100 from Cundinamarca Province, Colombia). **d–f** Basidiospores mounted in water (**d** KPM-NC 17827 [*RH30*]; **e** KPM-NC 17828 [*RH1181*; a potential hybrid specimen between *O. potteri* and a closely related unknown species]; **f** holotype [MICH 12376]). **g** Basidiospores mounted in lacto-glycerol after pre-soaking in 3% KOH and water (*MES807* [FLAS-F-66548]). **h** Basidiospores under SEM (FLAS-F-62022). **i** Peridium (*MES807* [FLAS-F-66548]). Scale bars: **a**, **c** = 1 cm, **b** = 5 mm, **d**–**h** = 10 μm, **i** = 50 μm

*Basionym: Octaviania asterosperma* var. *potteri* Singer & A.H. Sm., *Mem. Torrey Bot*. *Club* **21** (3): 10 (1959).

*Type*: **USA:** *Michigan*: Ithaca, Gratiot, Schovence’s Woods on exposed soil along a logging road in rich heavy soil (mud), 17 Sep. 1949, *V. Potter 8898* (MICH 12376 – holotype).

*Description*: *Basidiomata* sequestrate, mostly 8–20 mm diam, firm, rubbery, subglobose, depressed-globose or reniform; surface smooth or floccose to minutely scaly, white at first then becoming ochraceous at maturity, initially turning red or sometimes greenish blue at the base when touched or injured, gradually turning black. *Peridium* varying in thickness, mostly not exceeding 0.8 mm thick; context white, showing the same pattern of discoloration as the peridial surface. *Gleba* brown at maturity, finally becoming blackish brown, composed of locules filled with brown basidiospores and whitish mycelial veins, typical of the genus. *Stipe* (sterile base) rudimentary to pulvinate, white, with white rhizomorphs at base. *Odour* fragrant at first, becoming pungent at maturity.

*Basidiospores* 9–13.4(− 14) × 7.6–12.3(− 13.2) μm, mean 11.2 × 9.9 μm (SD: 1.06 [length], 1.14 [width]), subglobose to broadly ellipsoid (*Q* = 1–1.42, *Q*_*m*_ = 1.13), dextrinoid, light yellowish brown to ochraceous brown, covered with coarse, large pyramidal spines 1.6–3.4 μm high and 1.6–4.5 μm wide with a single slit-like cavity inside; spore walls 1.4–2.2 μm thick, with a pedicel up to 13 μm long at the base. Basidia 19–35 × 10–15.5 μm, clavate, mostly 2-spored, rarely 3- or 4-spored. *Subhymenium* not well developed. *Tramal plates* 30–200 μm thick, hyaline or light yellowish brown, of subparallel to interwoven, inflated, hyaline, filamentous hyphae 3–15 μm broad. *Peridium* 200–450 μm thick, context hyaline or light yellowish brown, yellow-brown near the surface, of interwoven, inflated filamentous hyphae 3–13 μm broad, pseudoparenchymatous cells to 45 μm diam at maturity; cell walls ca. 1 μm thick; peridiopellis very thin (to 60 μm thick) or absent in some parts, of partially inflated, septate, filamentous hyphae 3–7 μm broad subparallel to surface. *Clamp connection*s absent in all tissues.

*Habitat, known distribution and seasonality:* Hypogeous or subhypogeous under species of *Fagaceae*; eastern North America (Canada [Quebec], USA [IA, IN, FL, MN, NC, WV]), East Asia (Japan [Hokkaido]), South America (Colombia); summer to autumn.

*Other specimens examined:* **USA:** *Iowa*: Boone Co., Ledges State Park, 20 Sep. 2007, under *Quercus alba*, *R. Healy RH3* (FH 00284316); Emmet Co., Fort Defiance State Park, under *Quercus rubra, Ostrya virginiana, Tilia americana*, 26 Jul. 2000. *R. Healy RH720* (ISC-F-0072478); Story Co., Ames, Inis Grove Park, under *Q. alba, O. virginiana, T. americana,* 18 Aug.1998, *R. Healy RH234* (ISC-F-0072476); Ames, YMCA Woods, under *Q. alba*, 21 Sep. 1996, *R. Healy* (ISC-F-0072477); ibid, 25 Jul. 1997, *R. Healy RH48* (ISC-F-0072479); ibid, 25 Aug. 1999, *R. Healy RH555* (ISC-F-0072471); ibid, 9 Aug. 2000, *R. Healy RH750* (ISC-F-0072473); ibid, 27 Aug. 2000, *R. Healy RH782* (ISC-F-0072475); ibid, 27 Aug. 2007, *R. Healy* (FLAS-F-62023); ibid, 25 Sep. 2006, *E. Braun and Mycology Class* (ISC-F-0072472); ibid, 6 Sep. 2007, *R. Healy RH30* (FH 00284311; duplicates in KPM-NC 17827 & FLAS-F-66562); Hickory Grove Park, on slope by man-made lake, under *Quercus macrocarpa* and *T. Americana*, 11 Aug. 2009, *R. Healy* (FLAS-F-62022); Van Buren Co., Lacey-Keosaqua State Park, under *Q. alba* and *T. americana*, 30 Jul. 2001, *L. McCormick* (ISC-F-0072474); *Indiana*, Fort Wayne, 7 Nov. 2014, *K. Parker MES806* (FLAS-F-66547); ibid, *K. Parker MES807* (FLAS-F-66548); *Minnesota*, Fillmore Co., Forestville State Park, in mixed oak woods, 5 Aug. 2009, E.G. *McLaughlin RH973* (MIN 912630); ibid, 10 Jul. 2010, *R. Healy RH1181* (KPM-NC 17828, duplicate in FLAS-F-66563); Rice Co., Nerstrand Big Woods State Park, in mixed oak woods, 8 Aug. 2009, *R. Estell RH997* (MIN 912622); Washington Co., Afton State Park, in mixed oak woods, 27 Aug. 2009, *R. Healy RH1017* (MIN912618); ibid, *D.L. McLaughlin RH1018* (MIN912635); *North Carolina*, McDowell Co., along Blue Ridge Parkway near Mineral Museum, 21 Sep. 2003, *T. Eliott Trappe 32742* (FLAS-F-66549); at junction of Jones and Onslow Co., Croatan National Forest, White Oak River, 17 Jul. 2007, *T. Elliot MES801* (FLAS-F-66550); *Florida*, Wakulla Co., Skipper Bay road, St Marks NW refuge, under *Pinus elliottii* and *Q. virginiana*, 29 Dec. 2003, *D. Mitchell & W. Roody DMEL04–18, Trappe 32178* (OSC 131925); *West Virginia*, Randolph Co., Stuarts Park, 19 Sep. 1999, *K. St. Louis Trappe 25908* (FLAS-F-66551); McDowell Co., Bervind Wildlife Management Area, 11 Jul. 2002, *D. Mitchell Trappe 27950* (FLAS-F-66773); **CANADA:** *Quebec*, Montreal, 13 Sep. 1991, *F. Marzitelli Trappe 12445* (FLAS-F-66546); **JAPAN:** *Hokkaido*, Tomakomai City, near Kuchinashi-numa Pond, under *Quercus crispula*, 12 Sep. 2011, *M. Ohmae* (KPM-NC 18032); ibid, 21 Sep. 2012, *K. Yamamoto & T. Orihara* (KPM-NC 25043); **COLOMBIA:** *Cundinamarca Province*, Guacheta, Reserva Natural el Chaute o Robledal, Road from Guacheta to Raquira km 6, under *Quercus humboldtii,* 28 Feb. 2020, *A. Corrales 1036* (HUA 222100).

*Remarks*: *Octaviania potteri* was originally described from a specimen from Michigan, USA (Singer and Smith 1960). North American specimens have been reported from Quebec province in Eastern Canada and six states in eastern North America (NA). However, this taxon has not previously been collected in western NA despite extensive truffle research in California and the Pacific Northwest (e.g. Gilkey 1954; Trappe and Castellano 2000; Trappe et al. 2009). Interestingly, this species shows a remarkable disjunct distribution between eastern North America, South America (Colombia) and East Asia (Japan) (Fig. 1). This is the broadest distributional range of any known *Octaviania* species. As far as we know, this is also the first record of *Octaviania s. str.* from South America. The dispersal mechanism of *O. potteri* individuals is worth future investigation from a phylogeographical viewpoint. Morphologically, it is difficult to characterize this species because most characters of the basidiomes are typical of other taxa in the subgenus *Octaviania*. However, the distinctive dextrinoid reaction of the basidiospores and the very thin peridiopellis (i.e. an outermost filamentous layer of the peridium) that is sometimes absent in patches are two features that are distinct compared to any of the closely related species.

## DISCUSSION

Europe was considered the centre of biodiversity of *Octaviania* since the first description in 1831 (e.g., Hesse 1891; Pegler and Young 1979; Vittadini 1831), but Orihara et al. (2012a) revealed that this genus is also remarkably diverse in East Asia. Orihara et al. (2012a) also described 11 new species and one species that they provisionally named as “*Octaviania* sp. E”. Our study reinforces the high species diversity of *Octaviania* in East Asia by proposing two additional new species, *O. tenuipes* (i.e. “*Octaviania* sp. E”) and *O. tomentosa*. Our studies also revealed the new status of *O. potteri*, which was previously known only from eastern North America (Singer and Smith 1960) and is shown here to also occur in East Asia (Japan) and in South America (Colombia). Taking these new results into account, approximately half of the known species of *Octaviania* can be found in Japan (i.e. 14 species). In contrast, only four species are known from North America (Coker and Couch 1928; Orihara et al. 2012a; Singer and Smith 1960; Trappe and Castellano 2000) and approximately six species are currently recognized from Europe (Paz et al. 2016). These results suggest that Japan and the other regions of temperate East Asia are likely the centre of diversity for the genus *Octaviania*.

Notably, we also found that the distribution of *O. potteri* extends to a montane dry forest in Colombia dominated by the ectomycorrhizal host tree *Quercus humboldtii*. This is the first record of a true *Octaviania* from South America. Horak (1964) described *Octaviania chilensis* from Chile, but this species was later transferred to *Stephanospora* in *Agaricales* (Vidal 2004). Species of *Octaviania* subg. *Octaviania* are always associated with *Fagaceae*, and Colombian *Q. humboldtii* is considered to have migrated from Central America via the Isthmus of Panama in the Middle to Late Pleistocene (van der Hammen 1974). Thus, it is most likely that *O. potteri* migrated along with *Q. humboldtii,* the only oak species native to South America.

Generic relationships in the *Leccinoideae*, particularly among the genera *Leccinum*, *Leccinellum*, *Chamonixia, Octaviania, Rossbeevera,* and *Turmalinea*, have never been fully resolved with confidence in previous phylogenetic and systematic studies (e.g. Orihara et al. 2012a, 2016a; Wu et al. 2014, 2016). Kuo and Ortiz-Santana (2020) provided a large-scale, multilocus phylogeny that focused on epigeous *Leccinum* and *Leccinellum* species based on the nLSU, *TEF1* and *RPB2* regions. The resulting phylogenetic tree showed many polyphyletic clades of *Leccinum* and *Leccinellum s. lat.* within *Leccinoideae* and most of their phylogenetic relationships were unresolved. Accordingly, they synonymized the sequestrate genera *Chamonixia, Octaviania, Rossbeevera,* and *Turmalinea*, as well as epigeous *Leccinellum*, into a broadly circumscribed genus *Leccinum s.lat.* In our study, we incorporated the *RPB1* region into our multilocus dataset. The resulting trees provided robust phylogenetic support for most of the generic relationships in the *Leccinoideae*. The two exceptions were the lack of resolution in the branching pattern between *Chamonixia* and the other genera as well as the phylogenetic placement of some generic-level clades of epigeous *Leccinoideae* excluded from *Leccinum* and *Leccinellum s. str.* (i.e. *Leccinellum albellum*, *L. quercophilum* and *Leccinum violaceotinctum*; Fig. 1). This multilocus phylogeny also resolved most of the species-level relationships in *Octaviania*. This exemplifies the usefulness of *RPB1* for phylogenetic studies on *Leccinoideae* and highlights the fact that our multilocus phylogeny shows promise for resolving the genus-level relationships within the *Leccinoideae*.

Interestingly, we found that some of the *Octaviania* specimens had heterogeneous *RPB1* sequences compared to the other specimens of the same species. Comparison of the four single-gene tree topologies unexpectedly revealed that one *O. tenuipes* specimen (KPM-NC 27968) had an *RPB1* sequence identical to *O. japonimontana* (Fig. 2). Furthermore, the *RPB1* phylogeny showed that one *O. potteri* specimen from Minnesota (KPM-NC 17828; *RH1181*) was not placed within any of the known species-level clades in *O.* subg. *Octaviania*. Instead, this specimen formed a unique, phylogenetically distant branch (Fig. 2). These topological inconsistencies are best explained by interspecific hybridization between two closely related species.

*Octaviania tenuipes* consistently occurs in evergreen *Quercus* and *Castanopsis* forests in subtropical to temperate regions of Japan, whereas *O. japonimontana* occurs in deciduous *Quercus* and *Fagus crenata* forests in mountainous, temperate regions. The possible “hybrid” specimen between *O. tenuipes* and *O. japonimontana* (KPM-NC 27968) was collected in the Takakuma mountain range of Kyushu, Japan, which is known as a southern border of the distribution of *F. crenata*, the potential ectomycorrhizal host of *O. japonimontana*. Although we have not been able to find *O. japonimontana* in that mountain range, it is possible that the two closely related species inhabited the two adjacent vegetations and hybridized due to minimal putative reproductive barriers between the two species. This potential interspecific hybridization likely occurred recently because the “hybrid” *O. tenuipes* specimen (KPM-NC 27968) has a conserved *RPB1* sequence of *O. japonimontana* (i.e. 100% identical to other *O. japonimontana* sequences). The hybrid nature of this specimen was also supported by the network analysis based on the combined dataset of the ITS, nLSU, *RPB1* and *TEF1* regions (Fig. 3). Stukenbrock (2016) summarized that when two allopatric, fungal species come into contact they more readily hybridized than sympatric species, referring to the case of *Neurospora* species (Turner et al. 2011). Leducq et al. (2016) clarified that one North American lineage in *Saccharomyces paradoxus* is an incipient, hybrid species resulting from secondary contact of two geographically isolated, allopatric lineages after the last glaciation. Similarly, Gladieux et al. (2011) showed that hybridization between two closely related European *Microbotryum* species tends to be induced by secondary contact following initial divergence in allopatry. Although genome-level genetic comparisons will be needed to verify a potential hybridization between *O. tenuipes* and *O. japonimontana*, our hypothesis of hybridization is supported by other cases of hybridization in fungi such as those discussed above.

Similarly, the heterogenous *RPB1* sequence in the *O. potteri* specimen from Minnesota (KPM-NC 17828 [*RH1181*]) is likely the result of another interspecific hybridization between *O. potteri* and an unknown North American species within subg. *Octaviania*. So far, no such species closely related to *O. kobayasii* has been described, but our results strongly suggest that there is another undescribed species in this lineage from North America. We assume that this unknown *Octaviania* sp. is sympatric with *O. potteri* or at least they have bordering distributions somewhere in the upper Midwest of eastern North America. Further collecting surveys for truffle-like fungi in this region may confirm the existence of this unknown *Octaviania* species in the future.

In addition, the distinct intraspecific divergence between the *O. japonimontana* KPM-NC 26723 specimen and the other specimens in the *TEF1* phylogeny could be an additional signature of past intraspecific hybridization (Figs. 2, 4g). This topological pattern is similar to that of *O. potteri* in the *RPB1* phylogeny, although the genetic distance between the two lineages is less in the case of *O. japonimontana*. Therefore, it is likely that an unknown intraspecific lineage genetically distant to the core *O. japonimontana* clade exists or existed in the recent past around the habitat of the KPM-NC 26723 specimen. Geographically, the site where this specimen was collected was only about 250 m away from where another specimen (KPM-NC 26722) was collected on the same day, and we did not find any clear morphological differences between these specimens. Another possible cause of the topological differences is unusually accelerated molecular evolution of the KPM-NC 26723 specimen, but no such clear divergence was recognized in the other three DNA regions and the rate of divergence in the *TEF1* tree seems stable in other species-level clades (Fig. 2). Therefore, accelerated evolutionary rates seems less likely than hybridization.

Intra- and inter-specific hybridization in fungi has been frequently documented in plant pathogens (e.g. Brasier et al. 1999; Depotter et al. 2016; Feurtey et al. 2019; Newcombe et al. 2000; Schardl and Craven 2003; Stukenbrock 2016), yeasts (e.g., Gostinčar et al. 2018; Kuehne et al. 2007; Leducq et al. 2016; Marcet-Houben and Gabaldón 2015) and morels (*Morchella* spp.) (Du et al. 2016, 2019, 2020) but has been rarely reported in mushroom-forming basidiomycetes (Anderson et al. 1980; Stenlid and Karlsson 1991). Orihara et al. (2016a) illustrated that the frequent topological incongruences among gene trees of the sequestrate bolete genus, *Rossbeevera*, which is closely related to *Octaviania*, were likely to be derived from intraspecific gene introgression as well as incomplete lineage sorting (ILS). As far as we know, however, the present study is the first case that demonstrated interspecific hybridization in sequestrate basidiomycetes based on molecular evidence. Given the multiple traces of introgression among intraspecific lineages shown by Orihara et al. (2016a) and the interspecific hybridization within *Octaviania* discussed here, sequestrate genera in the *Leccinoideae* may be less reproductively isolated when the two lineages have been ecologically isolated (e.g. there is therefore no need for reproductive isolation to reinforce species boundaries). The precise mechanism of these interspecific hybridizations has not been discovered and the ploidy of putative hybrid specimens should be examined based on genome-level comparisons and analyses of chromosomes. We nevertheless suppose that interspecific hybridization may have promoted high genetic diversity within the sequestrate genera of the subfamily *Leccinoideae*.

## CONCLUSION

The multilocus phylogeny provided a robust phylogenetic framework of our study and revealed the phylogenetic placement of two new *Octaviania* species, *O. tenuipes* and *O. tomentosa,* collected from Japan. We also reclassified *O. asterosperma* var. *potteri* as an independent species, *O. potteri* stat. nov. This species exhibits an unusually broad range of distribution (i.e. North America, Japan, and Colombia), and this is the first record of *Octaviania* from South America. Comparison of the four single-gene tree topologies revealed remarkable topological inconsistencies within subgenus *Octaviania*, which are probably caused by inter- and intra-specific hybridization between two phylogenetically closely related lineages. Thus, we consider that these hybridization promote the high genetic and species diversity of *Octaviania*. Further genomic comparison among closely related species and precise population genetics will enlighten the speciation and diversification mechanisms within *Octaviania* and other sequestrate genera in the *Leccinoideae*.

## Supplementary Information

**Additional file 1: Fig. S1** ML tree of *Octaviania* subg. *Octaviania* based on nLSU dataset. Branches supported by both ML and BioNJ BS ≥ 75% are depicted as thickened black lines. Branches supported by either ML BS ≥ 75% or BioNJ BS ≥ 75% are shown as thickened gray lines. Statistical values below ML or BioNJ BS < 50% are not shown. Holotype materials are designated with asterisks (*). Two sequences of *Chamonixia caespitosa* were used for outgroups.

## Data Availability

Nucleotide sequences generated for this study were deposited in INSD via NCBI GenBank website (Table 1). The full alignments of datasets for phylogenetic analyses were submitted to TreeBASE and they are available under the following URL: http://purl.org/phylo/treebase/phylows/study/TB2:S26821.

## ABBREVIATIONS

ITS: Nuc rDNA internal transcribed spacer region
nLSU: Nuc rDNA large subunit (28S) region
*TEF1*: Translation elongation factor 1-α gene
*RPB1*: The largest subunit of RNA polymerase II gene
INSD: The International Nucleotide Sequence Databases
