# Supplementary figures and images for: Multilocus phylogenies reveal three new truffle-like taxa and the traces of interspecific hybridization in Octaviania (Boletaceae, Boletales)
Source: IMA Fungus. 2021 Jun 11;12:14. doi: 10.1186/s43008-021-00066-y (PMC8194053; doi:10.1186/s43008-021-00066-y)

LSU-rDNA phylogeny

ML BS  $\geq$  75%  
& NJ BS  $\geq$  75%  
Either  
ML BS  $\geq$  75%  
or NJ BS  $\geq$  75%

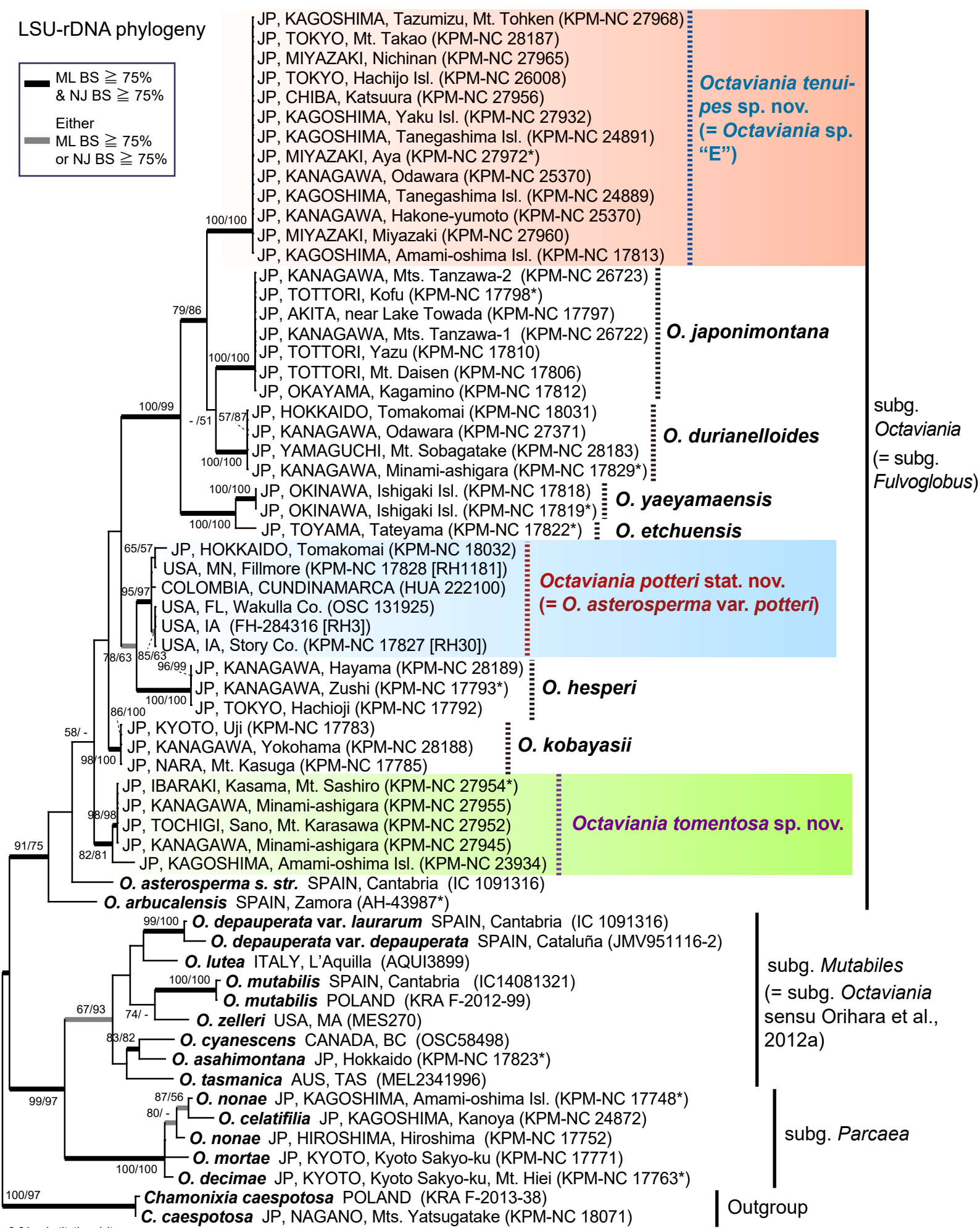

Supplement: Supplementary file 1 — Additional file 1: Fig. S1 ML tree of Octaviania subg. Octaviania based on nLSU dataset. Branches supported by both ML and BioNJ BS ≥ 75% are depicted as thickened black lines. Branches supported by either ML BS ≥ 75% or BioNJ BS ≥ 75% are shown as thickened gray lines. Statistical values below ML or BioNJ BS < 50% are not shown. Holotype materials are designated with asterisks (*). Two sequences of Chamonixia caespitosa were used for outgroups. [file 43008_2021_66_MOESM1_ESM.pdf]
